# First Complete Genome Sequence of Palo Verde Broom Emaravirus, Virus-Derived siRNA Signatures, and Phytohormone-Metabolite Profiling of Witches’ Broom-Affected Palo Verde Trees

**DOI:** 10.3390/v17081122

**Published:** 2025-08-15

**Authors:** Raphael O. Adegbola, Muhammad Ilyas, Dinusha C. Maheepala, Ursula K. Schuch, Judith K. Brown

**Affiliations:** School of Plant Sciences, The University of Arizona, 1140 E South Campus Drive, Tucson, AZ 85721, USA; roadegbola@arizona.edu (R.O.A.); milyassarwar@gmail.com (M.I.); dinusha1@arizona.edu (D.C.M.); uschuch@arizona.edu (U.K.S.)

**Keywords:** blue palo verde, *Emaravirus*, *Fimoviridae*, metabolites, palo verde eriophyid mite, *Parkinsonia florida*, phytohormones

## Abstract

Witches’ broom disease of blue palo verde (*Parkinsonia florida*) was reported more than sixty years ago. Characteristic symptoms consist of dense clusters of shortened, brittle branches and stunted leaves. The suspect causal agent has been identified as palo verde broom virus (PVBV), genus, *Emaravirus*, family, *Fimoviridae*. Here, the first complete PVBV genome sequence was determined, and virus small interfering RNAs (vsiRNAs), primary metabolites, and phytohormone profiles were characterized from infected palo verde leaves, adventitious shoots, flowers, and seeds. Based on pairwise distances, PVBV RNAs 1–4 shared 54–65% nucleotide identity and 19–51% amino acid similarity, respectively, with other emaraviruses, while PVBV RNA 5 shared no sequence homology with any emaravirus. The 21–24-nt virus-derived vsiRNAs, indicative of post-transcriptional gene silencing (PTGS), represented nearly the entire PVBV genome in flowers, leaves, seeds, and adventitious shoots; however, PVBV RNA 3 and RNA 4 were most heavily targeted in all plant parts. Evidence that six major phytohormones were altered in PVBV-infected compared to virus-free trees indicated that emaravirus-infected trees mount classical defense responses to virus infection and/or eriophyid mite infestations. Detection of PVBV RNA genome segments 1–5, accumulation of predominantly 21-nt vsiRNAs, homologous to the PVBV genome and transcripts, and altered levels of phytohormones and metabolites in PVBV-infected trees strongly implicate PVBV as the causal agent of witches’ broom disease.

## 1. Introduction

Palo verde trees (*Parkinsonia* spp.) are leguminous, drought-tolerant deciduous trees, classified in the genus *Parkinsonia* (syn. *Cercidium*), family *Fabaceae* [1,2,3]. Several species of palo verde are native to the Sonoran Desert, which spans the southwestern United States and northwestern Mexico [4,5,6]. In Arizona, the two native species of palo verde are blue palo verde, *Parkinsonia florida* (Benth. ex A. Gray) S. Watson and foothills palo verde, *P. microphylla* (Torr.) Rose & I.M. Johnston. In 1954, palo verde was designated as the Arizona state tree [7]. Several palo verde (*Parkinsonia*) species are sold as urban landscape trees, grown as large shrubs or multi-trunk trees, and are preferred trees for xeriscape landscaping in the low-elevation desert.

**Figure 1:**
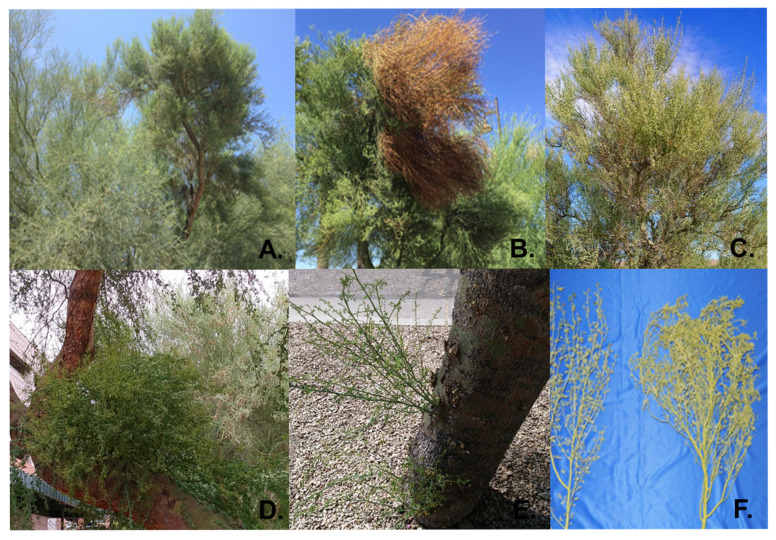
Symptoms of witches’ broom disease in the blue palo verde tree. Branch with large broom showing dense twig proliferation (**A**), broom dieback symptoms (**B**), multiple small brooms in tree canopy (**C**), broom emerging from the main trunk (**D**), adventitious shoots on the main trunk (**E**), and asymptomatic branch (left), and witches’ broom symptomatic branch (right) (**F**).

Since the 1940s, foliar symptoms have been associated with the naturally occurring ‘blue palo verde broom disease’ (BPVBD) [3,8]. Recently, BPVBD has become increasingly prevalent in nursery-grown palo verde trees in urban landscapes. Infected trees that have developed large, dense brooms in the canopy pose a danger when the branches break due to additional weight, especially during high winds and rain. The characteristic symptoms of palo verde broom disease are proliferation of dense twig growth, clusters of shortened, thornless branches, stunted leaves, and episodic mortality (Figure 1). Tip necrosis at the growing branches, or ‘dieback’, can occur following extreme deficits in annual precipitation, particularly when summer rainfall has been limited [1]. Although witches’ broom symptoms have been observed in native and urban blue palo verde trees in southern Arizona for nearly three-quarters of a century [8], it was not until recently when the palo verde broom virus (PVBV), genus, *Emaravirus* (order: *Bunyaviridae*; family, *Fimoviridae*), was identified in symptomatic trees by ‘discovery’ Illumina^®^ RNAseq that emaravirus etiology was suspected [9]. Although Koch’s postulates have not been completed, palo verde broom virus (PVBV) is considered the most likely causal agent of broom disease, based on RNAseq discovery sequencing [9] and detection by reverse-transcriptase PCR (RT-PCR) amplification in symptomatic but not asymptomatic trees [9,10].

Emaraviruses are plant viruses with a segmented, linear, negative-sense, single-stranded RNA genome that are transmitted by eriophyid mite species [11,12]. Since the discovery of the type species, *European mountain ash ringspot-associated virus*, thirty-three other emaravirus species, and presumed species, have been described [13]. Emaravirus particles are spherical and enveloped, and range in diameter from 80 to 200 nm. Infected cells are associated with electron-dense double-membrane-bound bodies (DMBs). Emaraviruses encode at least four and as many as ten negative-sense genomic RNA segments. Each segment encodes one open reading frame (ORF) in the complementary (+) sense. The RNA 1 genome segment is ~7.0 to 9.8 kb long and encodes the RNA-dependent RNA polymerase (RdRp), whereas RNA 2 is ~2.0 to 4.0 kb in length and codes for the glycoprotein precursor (GP). RNAs 3 and 4 are ~1.5 to 3.0 kb long and encode the virus nucleocapsid (NP) and movement protein (MP), respectively. The emaravirus RNA 5, 1.3 to 2.7 kb in length (present in all species), and RNAs 6–10, when present, are >~1.3 kb in length and encode proteins mostly of unknown function [11,14,15]. All emaravirus genome segments have a highly conserved, almost fully complementary 5′- and 3′- terminal end sequence of 19 to 23 nt that forms a base-paired structure referred to as a panhandle, which is involved in transcription, replication, and encapsidation. Both ends of each emaravirus RNA genome segment have a 13-nucleotide region that is fully conserved at the species level [16,17].

Emaraviruses can infect herbaceous and woody plant species [18,19,20,21,22,23,24,25,26], among which are maize and wheat, perennial forage species, fruit and forest trees, and ornamentals, spanning eudicot and monocot species and diverse climatic zones worldwide. In the United States, the high plains disease of maize and wheat is caused by high plains wheat mosaic virus (HPWMoV) [27], while fig mosaic virus (FMV) causes fig mosaic disease [28], rose rosette virus (RRV) is the causal agent of rose rosette disease [29], and blackberry leaf-mottle-associated virus (BLMaV) infects blackberry [30]. Thus far, BLMaV, RRV, and ti ringspot-associated virus (TiRSaV) occur only in the U.S. In contrast, HPWMoV and FMV are widely distributed in vegetatively propagated and cultivated plants throughout the world, including HPWMoV that infects wheat in Australia and New Zealand [31], and FMV in fig trees from China, Croatia, Montenegro, Russia, Syria, and Turkey [28,32].

Plant diseases can be caused by biotic and abiotic agents, and certain diseases that are caused by arthropod feeding and/or colonization cause symptoms that can be confused with plant pathogen etiologies [33], especially plant viruses. Plant virus infection and arthropod infestation of plants can trigger induction or suppression of core (primary) metabolites and phytohormones associated with plant defense responses [34]. Examples of primary metabolites and phytohormones, otherwise modulated according to growth stage in different organs during plant growth and reproduction [35,36,37], are ethylene (Et), jasmonic acid (JA), and salicylic acid (SA). They are also the most well-known plant signaling molecules involved in the induction of plant defenses to combat biotic and abiotic stress. Abscisic acid (ABA), auxin (Aux), cytokinin, and gibberellins (GA) are also essential for normal physiological functions in plants and can be triggered to induce defense responses [35,38,39]. Infection by plant viruses can alter signaling pathways and manipulate the physiology of the host plant, which in turn affects arthropod behavior [39,40,41]. Examples are enhanced plant volatile production, which can render virus-infected plants more attractive to the insect vector than virus-free plants [42,43], and increased or decreased longevity and/or reproduction in arthropods [40,44], which can benefit plant virus survival and increased virus transmission efficiency [45,46].

Nearly universally, plants respond to virus infection through a defense mechanism known as gene silencing or RNA interference (RNAi), which has evolved in eukaryotes to undermine virus replication by degrading virus-encoded transcripts and undermining other cellular mechanisms exploited during infection. For RNA viruses, the plant host activates or triggers RNA interference (RNAi) following recognition of viral dsRNA (replicative intermediate) and production of virus small interfering RNAs (vsiRNAs), 21–24 nucleotides in length [47,48]. Virus-induced RNA silencing involves initiation, amplification (RNA-dependent RNA polymerase, DICER nuclease activity (DCL)), and systemic spread of the signal. Silencing is initiated when viral dsRNAs are recognized by DCL-like ribonucleases to produce primary vsiRNAs. The siRNAs are loaded into the ARGONAUTE (AGO)-containing effector complexes to form the RNA-induced silencing complex (RISC), where they confer specificity for RNA or DNA targeting through a sequence homology-dependent mechanism. The association of the RISC with complementary target RNAs leads to cleavage, degradation, or translational inhibition of the cognate viral RNAs, whereas the interaction with target viral DNA causes modification of DNA and/or histones, resulting in transcriptional repression [49].

This study was carried out to determine and characterize the first complete genome sequence of PVBV by high-throughput sequencing (HTS). And, in lieu of completing Koch’s postulates for this disease of a woody perennial, which require mite colonization and virus transmission for development of characteristic broom symptoms, virus-induced small RNAs (vsiRNAs) (20–25 nucleotides) and phytohormone profiles were analyzed as substitutes of ‘pathogenicity indicators’. The latter experiments were designed to test the hypothesis that this pathosystem will trigger one or more host defense responses to PVBV infection and/or eriophyid mite colonization. Total RNA and/or soluble extracts were isolated from PVBV-infected and virus-free palo verde adventitious shoots, flowers, leaves, and seeds, and submitted for RNAseq Illumina sequencing, while plant extracts were analyzed for phytohormone content by high-performance liquid chromatography–mass spectrometry (LC-MS/MS).

## 2. Materials and Methods

### 2.1. Sample Collections

During 2015–2016, leaves, flowers, seeds, and adventitious shoots growing from the base of the tree trunk were collected from blue palo verde trees (n = 42). The trees were located at Reid Park Zoo, at the crossing of east Camino Campestre and south Via Golondrina streets, and at the University of Arizona campus, Tucson, at the Molecular Biology Building, northwest, and the Womens’ Studies Building, respectively. Samples were pooled and consisted of leaves from broom-asymptomatic, virus-free trees (P2), and broom-symptomatic trees (P3, P4), adventitious shoots (P5), flowers from broom-symptomatic trees (P8), and seeds from broom-symptomatic trees (P9). Plant samples were collected, flash-frozen in liquid nitrogen, and stored at −80 °C. Seeds were stored at 4 °C until processed.

### 2.2. Total RNA Isolation and Discovery Illumina Sequencing

Plant tissue (~50 mg) was homogenized with 1.0 mL Fruitmate™ (Takara Bio Inc., Mountain View, CA, USA) for 2 min in a Mini-Beadbeater (BioSpec Products Inc. Bartlesville, OK, USA) using the default settings. The homogenate was centrifuged (Eppendorf 5424R microfuge, Eppendorf North America, Enfield, CT, USA) at 12,000× g at 4 °C for 5 min, and the supernatant was transferred to a 2.0 mL microfuge tube followed by centrifugation, as described above. One mL of TRIzol LS reagent was added to 800 μL of supernatant and incubated at room temperature, mixed continuously on a mini-orbital shaker for 5 min (VWR, Radnor, PA, USA). Chloroform/isoamyl alcohol (24:1) was added (320 μL), and the contents were mixed for 5 min, followed by centrifugation for 15 min. The supernatant (1.0 mL) was transferred to a clean, sterile tube, and 1.0 mL of chloroform/isoamyl alcohol was added, gently inverted several times, and centrifuged for 5 min. The supernatant (~800 μL) was transferred to a clean, sterile tube, and total RNA was precipitated by the addition of an equal volume of isopropanol, incubated at −20 °C for 5 min, and centrifuged for 10 min. The pellet was washed with 75% ethanol, recovered by centrifugation, vacuum-dried and dissolved in 50 μL RNase-free water.

Total RNA (~2 μg) isolated from the respective palo verde samples was used to prepare large and small RNA cDNA libraries, from the following samples: leaves of asymptomatic, apparently virus-free trees (P2, n = 5), broom-symptomatic trees (P3, n = 14; and P4, n = 6), broom-symptomatic trees’ adventitious shoots (P5, n = 8), broom-symptomatic trees’ flowers (P8, n = 6), and seeds from broom-symptomatic trees (P9, n = 3). Library preparation and sequencing was outsourced (Roy J. Carver Biotechnology Center, University of Illinois, Urbana-Champaign, IL, USA).

### 2.3. Virus Genome Sequence Analyses

Adaptors were removed with BBDuk (parameters: ktrim = r k = 23 mink = 8 hdist = 1 tpe tbo barcodefilter chastityfilter), implemented in BBMap v38.96 [50]. The quality of reads was analyzed using FastQC v0.11.9 [51] and trimmed using “Trim Galore” (https://www.bioinformatics.babraham.ac.uk/projects/trim_galore/, accessed on 1 August 2022) to remove low-quality reads. Filtered reads were assembled de novo using Trinity v2.4.0 [52] and SPAdes 3.15.5 [53]. Virus-specific de novo contigs were selected using stand-alone BlastX and BlastN search tools [54]. Virus-specific de novo contigs were deposited in the NCBI GenBank database as the accession numbers MF766024-MF766043 and OM250026-OM250030.

The small RNAs were analyzed by reference-based mapping, based on the specific plant sample type. The virus-specific de novo contigs were used as reference sequences to map small RNA-seq reads using Bowtie v1.3.1 [55] on the Linux-based High-Performance Computing (HPC) cluster, the University of Arizona. The Sequence Alignment Map (SAM) output files were converted to Binary Alignment Map (BAM) format, sorted, and indexed. The mapped reads were recovered (-F 4), and based on strand bias, assigned as positive-strand (-F 16) or negative-strand virus RNA (-f 16) using SAMtools [56]. The sequence length distribution and strand bias plots were made using the viRome package [legacy] [57] in R v4.2.1 [58], respectively.

To identify the PVBV genomic targets of siRNAs, reads 20 to 24 nt in length were isolated from the respective pooled samples, i.e., P2 through P5, P8, and P9, using an in-house-developed script (available on request), and mapped against virus-specific de novo assembled contigs, as described above. The number of siRNAs mapped to each site on the positive or negative strand was calculated using Bedtools v2.29.2 (genomecov-bg-strand + or −) [59]. To scale data to counts per million (cpm), the number of mapped reads in each pool was divided by the total number of 20–24 nt reads per pool and multiplied by 1000,000 (in-house script). The normalized data were visualized in Circos [60], and Sush i.R (plotBedgraph) [61] in R v4.2.1 [58]. Bar graphs illustrating the normalized counts were drawn in the ggplot2 package [62], in R v4.2.1 [58].

Virus open reading frames (ORFs) were predicted using NCBI ORFfinder [63]. The molecular weight of predicted amino acid sequences of virus-encoded proteins was estimated using the isoeletric point/molecular weight computational tool available at the ExPASy server [64]. Transmembrane helices were predicted using TMHMM 2.0 [65], while the glycosylation sites were predicted using NetNGlyc 1.0 and NetOGlyc 4.0 [66], and cleavage sites were predicted using SignalP v. 5.0 [67]. The RNA secondary structure was modeled using PROMALS3D [68,69] and PSIPRED workbench [70].

Multiple sequence alignments were carried out using the Muscle algorithm [71]. The pairwise nucleotide sequence identity for each viral RNA segment was estimated by the Sequence Demarcation Tool (SDT) v1.3 [72]. Phylogenetic analysis (maximum likelihood) was carried out in CIPRES [73] using the Random Axelerated Maximum Likelihood (RAxML) method [74] and 1000 bootstrap replicates. The optimal model of rate distribution was determined as the gamma model. Phylogenetic trees were visualized using the interactive Tree of Life tool (iTOL) v6.4 [75].

### 2.4. Emaravirus Contigs Confirmation by Reverse Transcription-Polymerase Chain Amplification

To verify representative RNAseq discovery results, virus-specific primers were designed to amplify a fragment of each PVBV RNA genome segment, RNAs 1–5 (Table S1). Reverse transcription of total RNA from BPVBD symptomatic and asymptomatic BPV trees was carried out using either the High-Capacity cDNA Reverse Transcription kit (Applied Biosystems, Waltham, MA, USA) or SuperScript IV First-Strand Synthesis System (Invitrogen, Waltham, MA, USA), according to the manufacturer’s protocols. The cDNA synthesis was primed by the addition of the respective emaravirus RNA-specific primer, PDAP_213 [76]. Polymerase chain reaction (PCR) amplification was carried out using REDTaq^®^ ReadyMix™ (Sigma-Aldrich, St. Louis, MO, USA) with 2 µL of cDNA template. The RT-PCR conditions consisted of one cycle of initial denaturation at 2 min for 94 °C, followed by 35 cycles of 30 s at 94 °C, annealing at 55–57 °C for 30 s, and extension at 72 °C for 30 s, with a final extension at 72 °C for 7 min. Amplicons were ligated into the pGEM-T Easy plasmid vector (Promega Corp., Madison, WI, USA) and plasmids were transformed into chemically competent DH5α *Escherichia coli* cells. At least two colonies from each transformation event were selected and screened by colony PCR amplification using standard molecular biology protocols. Plasmids bearing an insert of the expected size were sequenced bi-directionally (Sanger).

### 2.5. High-Performance Liquid Chromatography-Mass Spectrometry of Plant Sample Extracts

The phytohormone and metabolite analysis of palo verde leaf samples was outsourced (Tissue Imaging, Metabolomics, and Proteomics Laboratory, Washington State University, Pullman, WA, USA). The respective plant samples were collected from asymptomatic, apparently virus-free trees, broom-symptomatic trees, and adventitious shoots. The samples (200 mg fresh weight) were transferred to a 1.7 mL microcentrifuge tube and extracted in 1.0 mL of Bieleski solvent (methanol:chloroform:formic acid:water (12:5:2:1, *v*/*v*/*v*/*v*) using TissueLyser II (Qiagen, Valencia, CA, USA) at a frequency of 27 Hz for 3 min, after the addition of two 2.8 mm steel balls. Briefly, samples were ultrasonicated for 3 min and stirred for 10 min at 4 °C. After centrifugation at 21,000 RPM at 4 °C for 10 min, the supernatant was collected and vacuum-dried. Dried supernatants were dissolved in 50 μL of the mobile phase solution, consisting of acetonitrile:water (5:95) with 0.1% formic acid, and analyzed by UHPLC-MS.

Phytohormone and metabolite identification were carried out using the LC-MS system described above and an ACQUITY UPLC HSS T3 1.8 μm, 2.1 × 100 mm column (Waters Corp., Milford, MA, USA) at a flow rate of 0.3 ml min^−1^, with linear gradients of solvent A (0.1% formic acid) and solvent B (0.1% formic acid in acetonitrile) with the following profile: 0 min, 5% B; 0.5 min, 5% B; 7.0 min, 50% B; 7.5 min, 95% B; 10 min, 95% B; 10.5 min, 5% A; and 14 min, 5% A. Data were collected in the negative ion mode using the source settings, as described above. The mass acquisition range and scan time were 50–600 Da and 0.5 s, respectively, and the collision energy ramp was 15–35 eV. Relative quantitation was determined by Progenesis QI (Nonlinear Dynamics, Waters Corp., Milford, MA, USA). Authentic standards were used for identification, and analyses were carried out using standard protocols. Hormone and metabolite graphs were made in ggplot2 [62], and principle component analysis biplots were made in MetaboAnalyst v6.0 [77].

## 3. Results

### 3.1. Genome Sequencing of Blue Palo Verde Witches Broom Disease-Associated Emaravirus

During the years 2015 and 2016, disease symptoms characterized by dense twig growth, stunted and thornless branches, and stem dieback were observed in tree canopies of palo verde trees throughout Tucson, AZ (Figure 1). To unravel the etiology of the disease, samples were pooled from different plant parts, as follows: leaves from broom-asymptomatic, apparently virus-free trees (P2) and broom-symptomatic trees (P3 and P4), adventitious shoots from symptomatic trees (P5), flowers from symptomatic trees (P8), and seeds from symptomatic trees (P9). High throughput sequencing (HTS) of total RNA yielded reads that ranged from 29,215,291 (P2) to 79,809,277 (P3) (Table 1).

The Illumina RNA-seq analysis identified five PVBV genome segments among the ~29.2 to ~79.8 million total reads. The greatest number of virus-specific reads were obtained from leaves, at ~0.58 to ~1.2 million (P3 and P4; Table 1), followed by flowers (P8) and seeds (P9) from symptomatic trees at ~0.45 to ~0.46 million (P8 and P9; Table 1). The fewest reads were obtained from the adventitious shoots (P5; Table 1), at 16,677.

Among PVBV RNA segments 1–5, RNA 3 was the most abundant, with reads ranging from 9128 in adventitious shoots (P5) to 737,004 in leaves (P4), accounting for 33% to 57% of the PVBV-specific reads (Table 1). RNA 4 was the second most abundant segment, with 4061 reads from adventitious shoots (P5) and 321,399 reads from leaves (P4), accounting for 15% to 25% of PVBV-specific reads (Table 1). The RNA1 and RNA2 segments were the third most abundant PVBV-specific reads, at 356,623 and 278,573, respectively. By comparison, RNA 5 was least abundant, at 193,383 reads.

Based on a Blastn search of the small RNA reads against the GenBank database, a ~7 kb sequence was identified that shared 63% similarity with RNA1 of HPWMoV, the closest PVBV relative, which is classified in the genus *Emaravirus*. Because well-studied emaraviruses have genomes consisting of variable numbers of RNA segments, from a minimum of 4 to a maximum of 10 [18,26,29,30,76,78,79], a Blastn search was carried out under variably stringent conditions to identify PVBV-specific contigs representing additional putative PVBV RNA genome segments, respectively. The searches identified five PVBV RNA segments, RNAs 1–5, of which RNA segments 1–4 shared the greatest sequence similarities with HPWMoV RNAs 1–4. The RNA 5 was unique among emaravirus RNA 5 segments and was exclusively associated with PVBV RNAs 1–4 recovered from palo verde trees. This result is consistent with previous studies that have reported that the RNA 5 segment shares no pairwise nucleotide identity or homology within the coding region with other emaraviruses [12,15].

**Figure 2:**
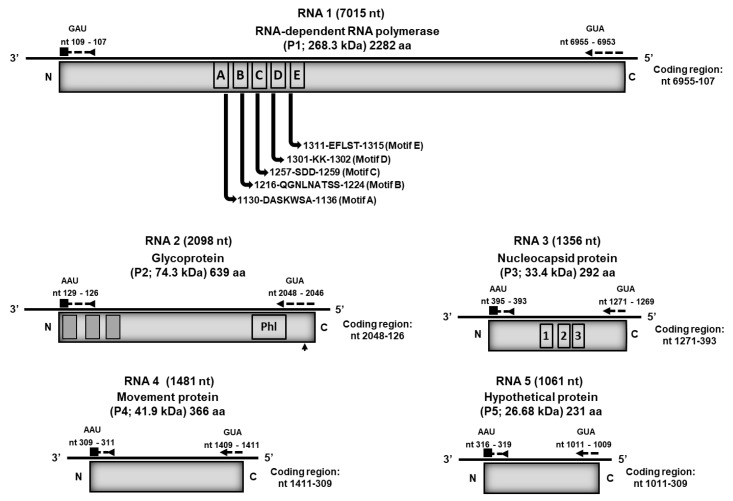
Genome organization of palo verde broom virus (PVBV) RNA segments 1–5. Conserved domain motifs, indicated by boxes and numbers, were identified in the PVBV RNA-dependent RNA polymerase (RdRp), glycoprotein (GP), and nucleocapsid protein (NP). Shaded boxes indicate GP transmembrane helices, and Phl indicates the phlebovirus glycoprotein motif. The top-facing bold arrow indicates a peptide cleavage site. The numbering of the nucleotide (nt) positions is based on the negative strand genomic RNAs. The numbering and placement of the motifs are diagrammatically represented based on the approximate amino acid positions and are not to scale.

A Blastn search of the contigs assembled from the RNA-seq libraries identified contigs analogous to HPWMoV RNAs 1–4 (GenBank database) in all broom-symptomatic samples. No HPWMoV analogous contigs were identified in RNA-seq libraries constructed from apparently virus-free, asymptomatic trees. Translation of the predicted open reading frames (ORFs) into the respective amino acid sequences showed that the predicted proteins were not truncated, indicating that the contigs represented complete coding regions. Based on amino acid (aa) sequence comparisons with the respective HPWMoV translated ORFS of HPWMoV, its closest relative, and other selected emaraviruses, used as reference sequences, the PVBV genome encodes an RNA-dependent RNA polymerase (RdRp) (RNA1), glycoprotein (GP) (RNA2), nucleocapsid protein (NP) (RNA3), movement protein (MP) (RNA4), and hypothetical protein (HP) (RNA5) (Figure 2).

### 3.2. RT-PCR Verification of Emaravirus Presence in Blue Palo Verde Tree Plant Tissue

The presence of PVBV RNA 1–5 assembled from reads was verified by RT-PCR amplification with viral RNA-specific primers (Table S1) and Sanger DNA sequencing, according to methods described above. Virus detection was carried out for leaves from broom-symptomatic trees (P4), adventitious shoots from symptomatic trees (P5), flowers from symptomatic trees (P8), seeds from symptomatic trees (P9), and leaves from broom-symptomatic trees, collected from trees at the different sites: Old Main #2 (OM2), Social Science East (SSE), and BPV18 greenhouse (BPV18-GH). Representative sequences for each sample type have been deposited in the NCBI GenBank database and assigned the accession numbers OM273626-40; Table S2. Alignment of the respective RNA1–5 genome sequences with de novo-assembled RNA1–5 contigs indicated that the PVBV genome segments recovered by RT-PCR amplification shared 99% identity with each respective de novo-assembled sequence.

### 3.3. Genome Organization of Palo Verde Broom Virus and Sequence Analyses

The genome of PVBV consists of five RNA segments (NCBI GenBank accessions MF766024-043; OM250026-30), with each segment composed of a single ORF that encodes one protein, which is consistent with other known emaraviruses (Figure 2). The five PVBV RNA segments had complementary ends of 20 to 23 nt in length and were highly conserved among the viral RNA 1–5 segments, as has been reported for other known emaraviruses. The RNA 1 to RNA 4c segments are known to constitute the essential or ‘core’ genome of emaraviruses, which encode proteins that shared aa sequence homology and recognizably similar motifs among known emaravirus species [12,29,80].

The PVBV RNA 1 is 7025 nt long and has an ORF located at nt coordinates GUA_6955−6953_ to GAU_109−107_ (Figure 2). This segment encodes the RNA-dependent RNA polymerase (RdRp, P1) protein consisting of 2282 aa residues and a molecular weight of 268.3 kDa. Pairwise nt and aa sequence comparisons indicated that they share 58.8–62.5% and 30.1–50.8% nt identity and aa similarity, respectively, with well-characterized emaravirus species (Table 2). The PVBV RdRp contains five predicted, highly conserved motifs (Figure 2) that correspond to core polymerase modules found in members of *Bunyavirales* [18,26,29,80]. Motifs A (DASKWSA_1130–1136_) and C (SDD_1257–1259_) are conserved among all emaraviruses, respectively, and form a portion of the palm domain of the replicase and binding sites for divalent metal cations [18,81]. Analogously, motif B, aa QGNXNX_2_S_2 1216–1224_, is conserved among emaraviruses and has predicted involvement in RNA binding, a function that is supported by the hypothesis that Gly_1217_ enhances motility of the peptide backbone [29]. Motif D (KK_1301–1302_) consists of Lys residues with predicted catalytic activity, given its’ close proximity to the Asp_1131_ residue in motif A, predicted by tertiary structure modeling [29]. Analogous to the conserved motif EFXSE in orthotospoviruses [82], motif E_1311–1315_ is conserved among emaraviruses, is implicated in cap-snatching among bunyaviruses, and has probable endonuclease activity [83]. Also, as in other emaraviruses, PVBV has the Bunyavirales-like endonuclease sequence domain, RHD_114–116_X_35_PD_152–153_X_14_EVK, that by analogy is involved in cap-snatching [84,85].

The PVBV RNA 2 genome segment is 2107 nt in length. The RNA 2 has one ORF located between the coordinates GUA_2048−2046_ to AAU_129−126_, which encodes the putative glycoprotein (GP; P2) consisting of 639 aa residues with a predicted molecular weight of 74.3 kDa (Figure 2). The PVBV RNA 2 sequence shares 57.5–61.3% pairwise identity with the RNA 2 found in other emaraviruses, and the PVBV P2 protein shares 21.4–40.6% aa similarity with the P2 protein encoded by known emaravirus species (Table 2). In silico analyses identified three transmembrane helices in P2, located at aa residues Arg_7_-Thr_29_; Phe_119_-Try_138_; and Try_181_-Ala_200_ (Figure S1a). In addition, three predicted N-glycosylation sites were identified at aa residues Asn_239_, Asn_340_, and Asn_401_. A peptide cleavage site was predicted at aa residues ISG22**↓**I23Y, with a probability of 0.4038 (Figure S1b). Also consistent with other emaravirus species, the PVBV P2 protein contains a conserved characteristic phlebovirus glycoprotein motif, GCX_2_CX_2_G_486–493_ (Figure 2).

PVBV RNA 3 is 1369 nt in length, has one ORF located between nucleotide coordinates GUA_1271−1269_ to AAU_395−393_, and encodes the putative nucleocapsid (NP; P3) protein (292 aa residues) with a molecular weight of 33.4 kDa (Figure 2). The RNA 3 and P3 protein shared 54.4–65.0% pairwise nt identity and 21.0–35.2% aa sequence similarity, respectively, with other known emaravirus species (Table 2). Also, PVVB P3 contains three conserved regions, NAVSX_2_RX_2_A_107–116_; NXLA_158–161_; and GXEF_179–182_, which is consistent with the P3 found in other known emaraviruses [81] and posited to participate in RNA binding [15].

PVBV RNA 4 is 1481 nt in length, has one ORF located at coordinates CAU_1411−1409_ to AAU_311−309_, encodes the putative viral movement (MP, P4) protein comprising 366 aa residues, and has a predicted molecular weight of 41.9 kDa (Figure 2). Nucleotide and aa sequence comparisons of the PVBV RNA 4 nt and P4 aa sequence with other emaraviruses showed that RNA 4 shares a nt sequence identity of 57.0–63.7% with known emaraviruses, whereas the P4 protein shares 19.0–51.7% aa sequence similarity with the P4 of other emaraviruses (Table 2). In silico analyses of the P4 protein identified conserved motifs, DXR_143–146_ and WKT_232–234_, which are found in the P4 of other known emaraviruses [86]. Also, the PVBV P4 protein was found to have a core secondary structure shared among viral movement proteins belonging to the 30 K superfamily [87] (Figure S2).

The PVBV RNA5 segment is 1061 nt in length and has one ORF located at coordinates CAU_1011−1009_ -UUA_309–311_ (Figure 2). The ORF encodes a hypothetical protein, P5, comprising 231 aa residues, and a predicted molecular weight of 26.7 kDa. A comparison of the PVBV P5 aa sequence with other emaravirus P5 proteins indicated 15.2 to 29.3% shared aa sequence similarity (Table 2). A comparison of PVBV P5 with other emaravirus P5 proteins revealed no identifiable conserved regions among them; however, consistent with other known emaraviruses, it is composed of mostly of alpha-helices [88]. The function of emaravirus P5 protein is unknown [11]. PVBV did not encode P7 and P8 proteins, like those associated with HPWMoV, which have been characterized as virus suppressors of host gene silencing [89]. The latter is consistent with the hypothesis that HP are recognized functional orthologs with roles in pathogenicity [30].

### 3.4. Phylogenetic Relationships of Palo Verde Broom Virus with Known Emaravirus Species

**Figure 3:**
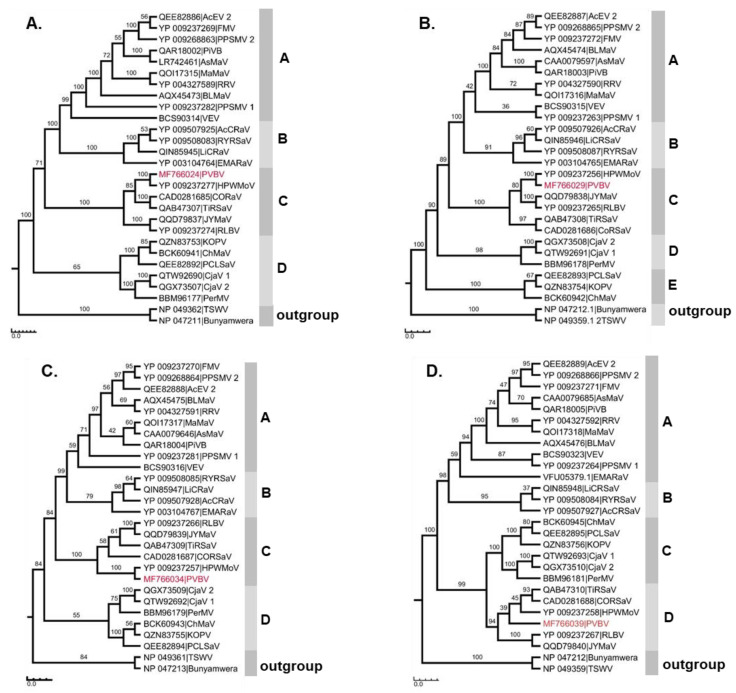
Phylogenetic analyses of palo verde broom virus (PVBV) RNA 1–4 segments using amino acid sequences of P1–P4 proteins, and comparisons to the encoded proteins of other emaraviruses. (**A**) RNA-dependent RNA polymerase (P1, RdRp), (**B**) glycoprotein (P2, GP), (**C**) nucleocapsid protein (P3, NP), and (**D**) movement protein (P4, MP). The phylogeny was reconstructed using the Random Axelerated Maximum Likelihood (RAxML) method [74] with 1000 bootstrap iterations, general time-reversible (GTR) evolutionary model, and gamma (G) distribution for the rate variation among sites. The analysis was carried out with PVBV and the following emaravirus species: actinidia emaravirus 2 (AcEV2), aspen mosaic-associated virus (AsMaV), blackberry leaf mottle associated virus (BLMaV), camellia japonica associated virus 1 (CjaV1), camellia japonica associated virus 2 (CjaV2), chrysanthemum mosaic-associated virus (ChMaV), common oak ringspot-associated virus (CORSaV), European mountain ash ringspot-associated virus (EMARaV), fig mosaic virus (FMV), high plains wheat mosaic virus (HPWMoV), jujube yellow mottle-associated virus (JYMaV), Karaka Ōkahu purepure virus (KŌPV), lilac chlorotic ringspot-associated virus (LiCRaV), maple mottle-associated virus (MaMaV), pear chlorotic leaf spot-associated virus (PCLSaV), perilla mosaic virus (PerMV), pigeonpea sterility mosaic virus 1 (PPSMV1), pigeonpea sterility mosaic virus 2 (PPSMV2), pistacia emaravirus B (PiVB), raspberry ringspot virus (RRSV), raspberry leaf blotch virus (RLBV), redbud yellow ringspot-associated virus (RYRaV), rose rosette virus (RRV), ti ringspot-associated virus (TiRSaV), and vitis emaravirus (VEV).

A maximum likelihood tree (RAxML) was reconstructed using the amino acid sequences of the RdRp, GP, NP, and MP of PVBV and emaraviruses for which the four core genome segments were available. Two members of the order *Bunyavirales* were included as outgroups (Figure 3A–D). The results of phylogenetic analyses indicated that the phylogenetic tree topology was similar across all four RNA segment-encoded proteins, resolving four groups for all proteins, except for the GP that resolved five clades (Figure 3A–D). A phylogeny could not be reconstructed for the P5 protein because this emaravirus genome segment shares negligible interspecific aa similarity with other emaravirus.

All ten emaraviruses, referred to as clade I, clustered as a monophyletic group consisting of AcEV-2, FMV, PPSMV-2, PiVB, AsMaV, MaMaV, RRV, BLMaV, PPSMV-1, and VEV. Clade II contained four species, AcCRaV, RYRSaV, LiCRaV, and EMARaV, while Clade III contained PVBV and its closest relatives, JYMaV, RLBV, TiRSaV, CORSaV, and HPWMoV. The fourth clade, IV, contained six species, ChMaV, KŌPV, PCLSaV, CjaV-2, CjaV-1, and PerMV. Among well-studied emaraviruses, HPWMoV, which infects monocot hosts, was identified as the closest relative of PVBV.

### 3.5. Palo Verde Broom Virus-Derived Small RNA Profiles in Blue Palo Verde Plant Tissues and/or Organs

**Figure 4:**
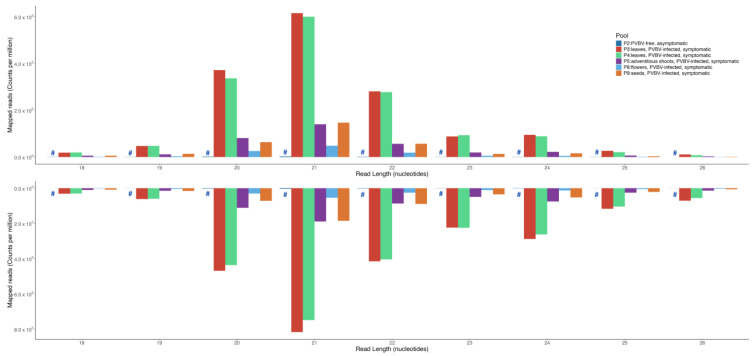
Size distribution profiles of genome and anti-genome strands of small interfering RNAs derived from different plant parts (leaf, adventitious shoot, flower, and seed) of palo verde broom virus (PVBV)-infected trees. The X-axis represents small RNA libraries from different plant parts. P2: leaves from broom-asymptomatic trees, denoted by ‘#’; P3–P4: leaves from broom-symptomatic trees; P5: adventitious shoots from symptomatic trees; P8: flowers from symptomatic trees; P9: seeds from symptomatic trees. The Y-axis represents total number of small RNA fractions mapped to the PVBV genome segments.

Small RNA reads were obtained from pooled RNA samples isolated from broom-asymptomatic, apparently virus-free trees (P2), broom-symptomatic trees (P3 and P4), adventitious shoots from symptomatic trees (P5), flowers from symptomatic trees (P8), and seeds from symptomatic trees (P9) (Table 3). The 20- to 24-nt reads were mapped to de novo-assembled, pooled PVBV RNA genome segments and normalized by dividing the number of mapped reads by the total number of reads per population. The different plant parts and/or organs accumulated variable levels of PVBV-derived vsiRNAs. The leaves yielded the most PVBV-derived vsiRNA reads, at 197,065.36 and 216,875.39 counts per million (cpm) for P3 and P4 treatments, respectively. This was followed by flowers at 110,337.29 cpm (P8), seeds at 77,302.41 cpm (P9), and adventitious shoots at 70,122.80 cpm (P5) (Table 3). The relative proportion of vsiRNAs, based on length, closely mirrored that of the entire small RNA population, i.e., virus- and plant-derived, in each pool (Figure S3), multiplying by 1,000,000 (i.e., counts per million). The 21-nt class of vsiRNAs was the most abundant, followed by the 20-nt, 22-nt, 24-nt, and 23-nt classes, respectively (Figure 4).

**Figure 5:**
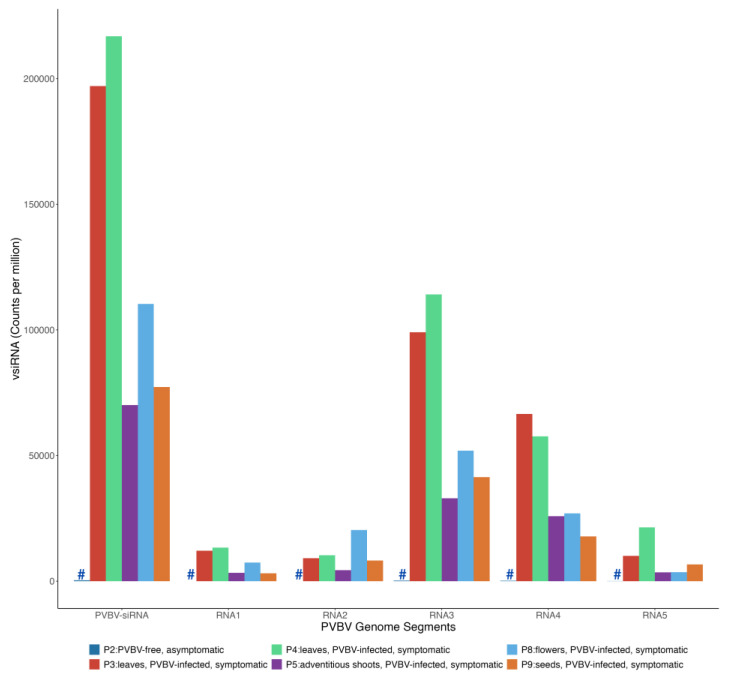
Accumulation of palo verde broom virus (PVBV) small interfering RNAs (vsiRNAs) across the individual RNA genome segments recovered from the different plant parts (leaf, adventitious shoot, flower, and seed) of palo verde broom virus (PVBV)-infected trees. The X-axis indicates total palo verde broom virus-specific vsiRNAs homologous to the PVBV genome and to each individual RNA segment. The Y-axis indicates the total number of vsiRNAs, expressed in counts per million (cpm), from the individual RNA genome segments that mapped to the PVBV genome. The small RNA libraries produced from different palo verde plants parts were as follows: P2: leaves from broom-asymptomatic trees, denoted by ‘#’; P3–P4: leaves from broom-symptomatic trees; P5: adventitious shoots from symptomatic trees; P8: flowers from symptomatic trees; P9: seeds from symptomatic trees.

**Figure 6:**
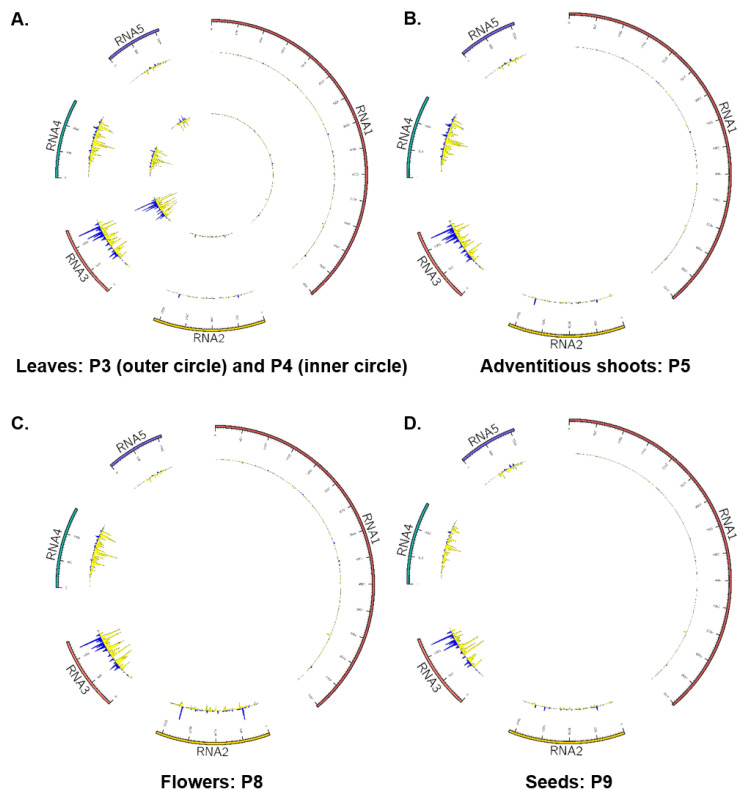
Genome coverage of palo verde broom virus (PVBV) small interfering RNAs (siRNA) mapped across the virus genome, showing the relative accumulation and ‘hotspots’ of PVBV-derived siRNAs across each individual RNA genome segment. The RNA 3 genome segment was the most heavily targeted by the post transcriptional gene silencing mechanism of the host plant. (**A**) Leaves from symptomatic trees—pool 3 and pool 4; (**B**) pool 5; (**C**) flowers—pool 8; and (**D**) seeds—pool 9. (**A**) The PVBV genome segment locations and approximate sizes (lengths). (**B**) Isolate P3, and (**C**) isolate P4.

When the proportion of vsiRNAs that mapped to each PVBV genome segment was quantified (Figure 5), the results were found to follow a trend similar to that of total RNA-seq reads per virus genome segment (Table 2), fostering robust confidence in the data. In symptomatic tissues/organs, the majority of the vsiRNAs mapped against RNA 3, and next, to RNA 4 (Figure 5, Figure 6 and Figure S5 and Table 3). A negative-strand bias was observed for the 21-, 22- and 24-nt siRNAs in all of the organ and tissue types analyzed (Figure S4).

To determine the specific mode of targeting of the PVBV genome segments by vsiRNAs in the different types of plant samples, strand-specific mapping was carried for each isolate separately, except for the P3 and P4 samples, which were pooled. For all samples, independent of plant part, RNA 3 was the most targeted genome segment by host RNAi mechanisms. While for RNA 1 and RNA 2, vsiRNA target sites (i.e., highest vsiRNA coverage) occurred throughout all genome segments, the majority of target sites for the RNA 3, 4, and 5 segments were most abundant near the 3′-terminal end of each of the segments (Figure 6A–D and Figure S5A–T), potentially indicative of vsiRNA processing mechanism(s).

### 3.6. Variation in Phytohormones and Metabolites in Leaves of Asymptomatic, Apparantly Virus-Free Trees, Broom-Symptomatic Trees, and Adventitous Shoots from Broom-Symptomatic Trees

**Figure 7:**
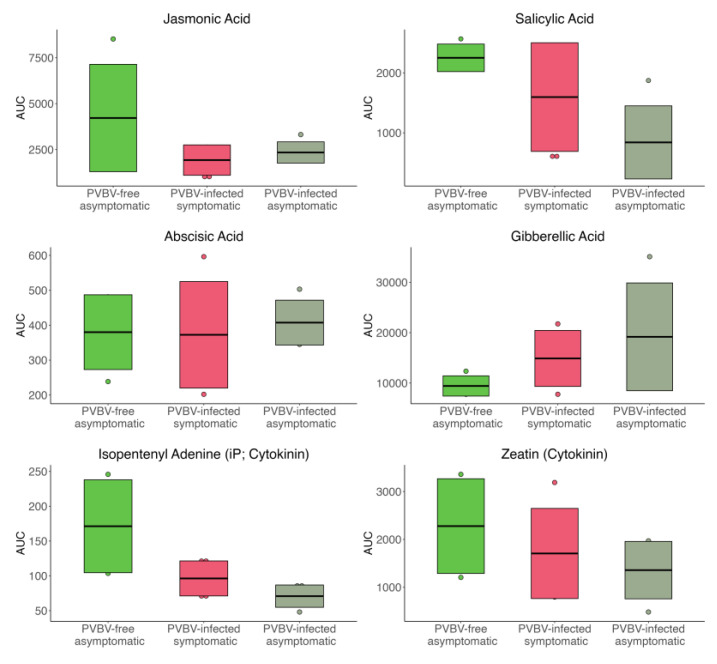
Levels of six phytohormones in leaf tissue collected from PVBV-free, asymptomatic trees, PVBV-infected, symptomatic (broom) shoots, and PVBV-infected, asymptomatic (adventitious) shoots. The Y-axes represent the area under the curve (AUC). The mid-, upper-, and bottom-crossbars of the box plots indicate averages and positive and negative standard deviations (SDs), respectively. The datapoints outside SD boundaries are marked by dots.

The abundance, indicated as the area under the curve, of six phytohormones, jasmonic acid (JA), salicylic acid (SA), abscisic acid (ABA), gibberellic acid (GA), and the cytokinins isopentenyladenine (iP) and zeatin, in leaf samples from asymptomatic and apparently virus-free trees, broom-symptomatic trees, and adventitious shoots from symptomatic and virus-infected trees are summarized in Figure 7. Only the iP levels contributed to a statistically significant difference between PVBV-free and PVBV-infected, asymptomatic trees. It should be noted that the variations in mean-area-under-the-curve (AUC) estimates reported could be associated with undefinable physiological phenomena, given the minuscule amounts of material required for the analyses. The average abundance of JA, SA, and cytokinins was relatively higher in virus-free leaves compared to virus-infected broom branches and adventitious shoots. Also, the mean JA level was lower in the broom branches than the adventitious shoots. The opposite pattern was observed for SA and cytokinin levels. The GA levels were the lowest in virus-free leaves, and highest in the asymptomatic, adventitious shoots from PVBV-infected trees. In contrast, the ABA levels did not fluctuate significantly between any of the tissue types, regardless of virus presence or absence.

**Figure 8:**
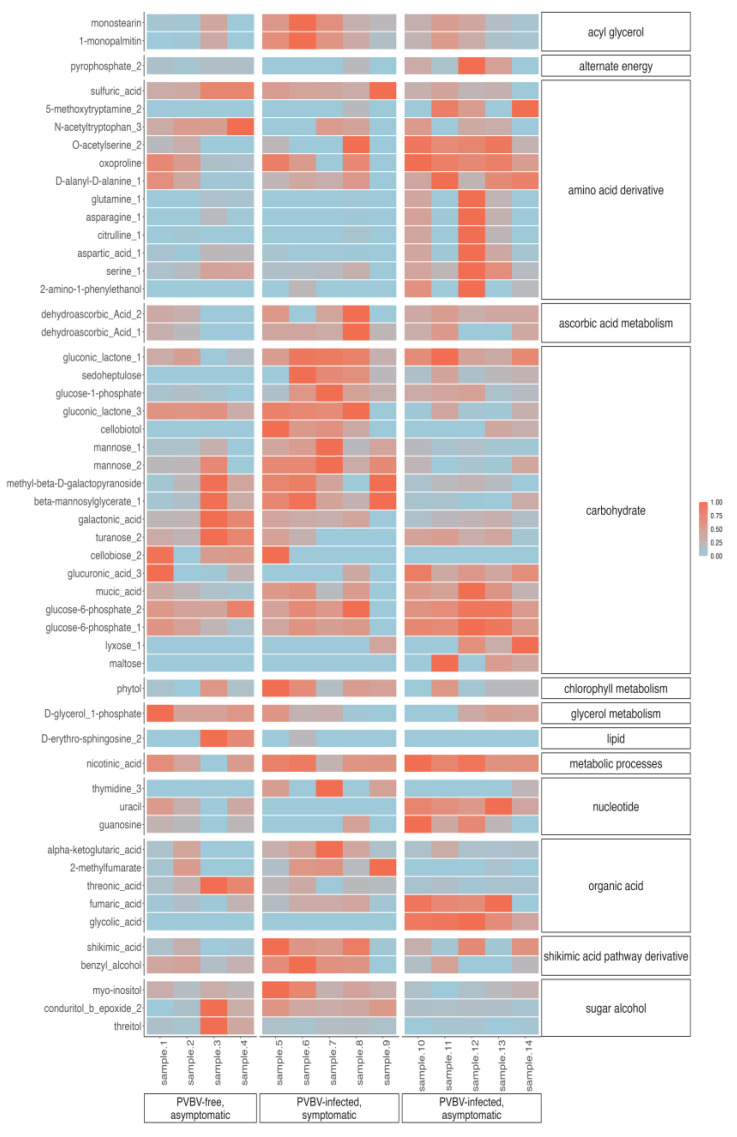
Normalized area-under-the-curve values for the top 50 metabolites (ANOVA) in leaves from PVBV-free, asymptomatic trees, PVBV-infected, symptomatic (broom) shoots, and PVBV-infected, asymptomatic (adventitious) shoots. Metabolites and functional categories are given on the left and right Y-axes, respectively.

Detectable changes in primary metabolic profiles with potential relevance to the study system were analyzed with respect to the top 50 metabolites, as determined by ANOVA. The results were summarized in heatmaps and PCA biplots to visualize the variation observed for selected metabolites associated with different paloverde tissues/organ samples (the same samples used for the phytohormone study). Results indicated that among the top 50 metabolites were sugar alcohol, shikimic acid pathway derivative, organic acid, nucleotide, lipid, glycerol metabolism, chlorophyll metabolism, carbohydrate, ascorbic acid metabolism, amino acid, and acyl glycerol categories (Figure 8 and Figure S6A,B), which were further enriched in either PVBV-free trees, broom shoots, or adventitious shoots. The adventitious shoots were enriched for nucleotide (e.g., uracil) and amino acid derivative (e.g., glutamine) categories, and pyrophosphate 2, relative to PVBV-free and broom shoots. Leaves collected from palo verde broom shoots contained a relatively high abundance of metabolite categories representing a sugar alcohol (e.g., myo-inositol), the shikimic acid pathway (e.g., shikimic acid), and chlorophyll metabolism (e.g., phytol), in relation to PVBV-free and adventitious shoots. Leaves from both broom-symptomatic (PVBV-infected) and adventitious shoots of virus-infected trees were enriched for categories representing an organic acid (e.g., alpha ketoglutaric acid), a carbohydrate (e.g., glucose-6-phosphate), ascorbic acid metabolism (e.g., dehydroascorbic acid) acyl glycerols (e.g., monostearin), and sulfuric and nicotinic acids, compared to PVBV-free leaves. By comparison, virus-free palo verde leaves were enriched for lipid (e.g., D-erythro-sphingosine) and glycerol metabolism categories (e.g., D-glycerol-3-phoshate), compared to leaves from PVBV-infected trees.

## 4. Discussion

In this study, the complete genome sequence of an elusive plant virus, palo verde broom virus (PVBV), recently associated with eriophyid mite-infested BPV trees exhibiting witches’ broom symptoms, was characterized from blue palo verde trees in Arizona using Illumina^®^ RNAseq technology. Comprehensive analyses, consisting of de novo assembly, RT-PCR amplification and confirmatory sequencing, pairwise distance and phylogenetic analysis, and nucleotide and amino acid sequence comparisons, have established that the PVBV genome consists of five RNA segments with a similar genome organization to other members of the genus *Emaravirus*, family *Fimoviridae*.

Emaraviruses are transmitted by at least one species of eriophyid mite that is generally host-specific [11,13]. The palo verde mite *Aculus cercidii* (Keifer, 1965) is the mite suspect vector of PVBV based on the observation that it is the most abundant common mite found on palo verde tree leaves and buds (personal communication, Drs. R. Ochoa and A. Ulsamer, Systematic Entomology Laboratory, USDA-ARS, BARC-West, Beltsville, MD, USA). This is consistent with previous reports of abnormalities in palo verde trees infested with eriophyid mites referred to as witches’ broom [90], prior to the discovery of the association of PVBV with blue palo verde trees. Although many eriophyid mites have been associated with abnormal growth in desert landscape plants [90], few if any studies have been conducted to investigate the possibility for emaravirus etiology, given other similar pathologies of known emaravirus causality. Among the best-studied emaravirus–mite vector complexes are fig mosaic virus (FMV; *Aceria ficus* Cotte), HPWMoV—*Aceria tosichella*, pigeon pea sterility mosaic virus (PPSMV; *A. cajani*), and others [11,28,91]. The apparently narrow host range of emaraviruses corresponds to the narrow host specificity of the respective eriophyid mite species [12]. Although Koch’s postulates of causality have yet to be fulfilled, the evidence presented here, as well as information gained from a previous effort to identify the etiological agent by Illumina DNA sequencing, has identified PVBV as the only pathological agent consistently associated with eriophyid mite-infested symptomatic blue palo verde trees (this study).

Consistent with the multi-segmented characteristic of other emaraviruses that have as few as four and as many as ten genome segments, the PVBV genome consists of five RNA segments that each encode one ORF in the complementary strand [11]. The PVBV RNA 1 to RNA 4 predicted proteins are conserved and most closely related to the analogous four RNAs of other emaravirus species. As expected, the RNA 5 segment was unrelated to the analogous RNA and predicted proteins known for other emaraviruses that have at least five genome segments. This supports the hypothesis that emaravirus P5 is likely involved in pathogenicity enhancement [89]. Based on comparative aa sequence analyses with well-studied reference emaravirus-encoded proteins, the PVBV genome encodes four essential emaravirus or ‘core’ proteins, RdRp, GP, NP, and MP [29,30]. The RNA 5 of PVBV, and other RNA segments described by others, encodes a hypothetical protein of unknown function. Recent studies of HPWMoV hypothetical proteins P7 and P8 showed that they both function as RNA silencing suppressors [89]. In contrast, the P2 to P8 proteins encoded by RLBV showed no detectable RNA silencing activity; however, the P6 and P7 proteins were reported to be associated with pathogenicity [14]. Another investigation of emaravirus hypothetical proteins [76] reported some extent of aa similarity among the P5 proteins of FMV, PPSMV, RLBV, and HPWMoV. Despite the homology reported among emaravirus P1 to P4 proteins, emaravirus hypothetical proteins may have evolved uniquely, possibly having been influenced by host type and nuances of host plant PTGS mechanisms [42], in that viruses may exploit host resources to influence the host phenotype and vector behavior, which may favor eriophyid mite vector transmission [42].

Phylogenetic analyses of PVBV aa sequences with representative emaraviruses corroborates the placement of PVBV based on pairwise and phylogenetic analyses of the viral nucleotide sequences within the genus *Emaravirus*. The tree topologies were similar for the four virus proteins, grouping the emaraviruses into four to five main phylogenetic clades. Despite attempts to identify all genomic RNAs, additional sequence analyses could lead to the discovery of additional genomic segments [9,76,92]. As described, PVBV was most closely related to HPWMoV [9] irrespective of the predicted virus proteins considered. Both viruses belong to the same phylogenetic group, with the next closest relatives being JYMaV, RLBV, TiRSaV, and CORSaV. Despite their close evolutionary relationships, the host plants of the different respective emaraviruses are not close relatives botanically. Despite the range of plant hosts known to be infected by emaraviruses, as a virus family, the host range of individual species is narrow, probably because the host range of the virus is restricted by the host specificity of the eriophyid mite vector [93]. Other than HPWMoV, all emaraviruses that infect woody perennials are propagated either vegetatively (clonally) or by seed, creating a route for virus transmission during plant propagation of trees in plant nurseries.

Virus-specific siRNAs (vsiRNAs) that mapped to the genome differed for the particular RNA genome segment, inferred from vsiRNAs levels (Table 3), suggesting that expression of virus-encoded proteins differed depending on plant part/organ/tissue examined here (Figure 5 and Figure 6). From the virus diagnosis perspective, examining the relative read counts from different plant parts shows the probable distribution of the virus, the importance of collecting samples from the optimal plant part for detection, and significance of basing the detection assay on the most highly expressed virus gene to reduce false negatives (Figure 4 and Figure 5). In both HTS approaches, the RNA 3 segment occurred in the highest proportion of reads in symptomatic leaf samples compared to all other viral segments and plant parts examined, respectively. This observation is consistent with PCLSaV, for which the RNA 3 segment accumulated to the highest levels, compared to the other segments, in symptomatic leaf samples [79]. The structural nucleoprotein (NP) encoded by RNA 3 is required for encapsidation of viral RNA genomic segments and was most abundant among the other viral-encoded proteins. Consistent with RNAseq observations that corroborate the presence of all five PVBV RNAs, the analogous RNAs 1–5 were detected by RT-PCR amplification in leaves collected from both symptomatic and asymptomatic branches of trees exhibiting witches’ broom symptoms (Table S2, Figure 1).

Characterization of vsiRNA expression profiles from asymptomatic and symptomatic palo verde trees showed that PVBV is recognized by the host post-transcriptional gene silencing (PTGS) defense system, as observed for many other plant virus pathosystems [94,95]. The vsiRNA profiles clearly show the involvement of PTGS against PVBV infection in *P. florida*. The predominance of 21- and 22-fragment sizes observed in vsiRNA pools (Figure S5) is indicative of post-transcriptional cleavage of viral mRNA and, potentially, of single-stranded genomic RNA regions with secondary structure [96,97]. Hypothetically, in *P. florida*, processing dsRNA, synthesized by orthologs of *Arabidopsis thaliana* RNA-dependent RNA polymerase 1 (RDR1)/RDR6, into siRNA duplexes is carried out by the Dicer-like 4 (DCL4) ortholog, which produces 21-nt duplexes, and to some extent by the DCL2 ortholog, which is responsible for the 22-nt component, both of which as siRNA duplexes bind to Argonaute (AGO) family proteins in RNA-induced silencing complexes (RISCs) that target viral RNA for cleavage [96,97,98,99]. The number of siRNA mapped against each PVBV genome segment closely align with the relative estimate of expression for each virus RNA genome segment in the different plant tissue examined. Localized differences occurred between the negative and positive strands, an observation that has been previously reported [100,101] but is also possibly attributable to target sequence selectivity of DCL, which is known to fluctuate with respect to the number of siRNA duplexes diced along the length of a virus genome, and/or to uneven interactions with the resulting duplexes associated with AGO that can lead to degradation of those with low affinities. In addition, a negative-stranded bias was observed in the siRNA pools (Figure S4). However, a pattern such as this could be an artifact of cDNA library preparation, if one strand has been selected over the other during first strand synthesis [96]. Some studies support a strong strand bias among some RNA viruses due to incomplete transcription of one strand by RNA-dependent RNA polymerase or instability following siRNA duplex formation [102,103].

Thus far, eriophyid mites have been implicated as the exclusive vector of emaraviruses, either by direct experimental evidence, and/or because of their association with an emaravirus-infected plant species [11,18,21,27,30,104,105]. Though vector transmission has not been established for PVBV, the eriophyid mite *Aculus cercidis* has been consistently associated with palo verde trees exhibiting witches’ broom symptoms [106]. Mite counts in naturally infested leaves and branches show that the palo verde mite is consistently associated with witches’ broom-symptomatic PVBV-positive BPV trees, compared to few or no mites in asymptomatic trees. The epidemiological importance of *A. cercidi* in the natural spread and distribution of PVBV, broom symptom development, virus–vector biology, and other factors that may influence virus spread requires further study.

Phytohormone and metabolite profiles of leaves collected from asymptomatic, apparently virus-free trees and from broom shoots and adventitious shoots of symptomatic, virus-infected trees (Figure 7 and Figure 8) indicate that compounds regulating physiological processes in BPV trees are responsive to pathogen-mediated stresses. During 2015, when the palo verde tree samples were processed for phytohormone and primary metabolite analyses, the relationship between PVBV and palo verde broom disease was only suspected. Thus, adventitious shoots were collected only from broom-symptomatic trees, and other collections were based on the plant parts available (flowers, leaves, shoots, seeds) and the availability of broom-affected or apparently broom-free trees. Consequently, adventitious shoot samples from broom-free (virus-free) trees were not available for comparison with shoots collected from PVBV-infected, symptomatic broom tissue. Even so, it was possible to analyze at least some of the tissue types from diseased and disease-free trees and obtain interesting, statistically significant results.

Similar results have been reported in other plant–virus–vector pathosytems, including tomato–tomato yellow leaf curl virus–whitefly, tomato–tomato spotted wilt virus–western flower thrips, *Capsicum annuum*–tomato zonate spot virus–western flower thrips, and tobacco–cucumber mosaic virus–aphid interactions [107,108,109,110,111,112]. Here, BPV leaves collected from virus-infected, symptomatic (broom) and virus-infected asymptomatic (adventitious) shoots showed reduced jasmonic acid (JA) and salicylic acid (SA), key plant immunity hormones, compared to PVBV-free, asymptomatic trees (Figure 7). Corroborating these findings, cucumber mosaic virus (*Cucumovirus*) resulted in low JA levels in comparison to uninoculated plants [113]. The exogenous application of JAs resulted in reduced rates of infection by several RNA viruses [39], and JA-mediated defenses suppressed virus disease incidence in response to foliar applications of methyl jasmonate [114]. It has been reported that southern rice black-streaked dwarf virus (SRBSDV; Fijivirus) and rice stripe virus (*Tenuivirus*) may downregulate SA activity by inducing the expression of rice *Nuclear Factor Y* [115]. In palo verde leaf samples, the mean level of SA was higher for eriophyid mite-infested broom leaves, compared to virus-infected adventitious shoots on which mite populations were sparse or undetectable. This is consistent with the results of studies involving whitefly, *Bemisia tabaci* Genn., for which increased SA responsive gene expression and reduced JA-dependent RNA levels have been documented [116].

PVBV-free, asymptomatic palo verde leaves contained lower levels of gibberellins (GAs) compared to the actively elongating adventitious shoots (PVBV-infected, asymptomatic) and broom shoots (PVBV-infected, symptomatic) to some extent. Because GA is important for plant shoot and leaf elongation, higher levels in rapidly growing adventitious shoots likely reflect physiological processes in fresh growth. The spike in GA may have been facilitated by virus infection, as has been reported for tomato spotted wilt virus (*Orthotospovirus*) and SRBSDV, which directly interacts with gibberellin-insensitive dwarf2 (GID2), the ortholog of DELLA in rice, a repressor of GA signaling, and leads to its degradation [108,117,118]. An analogous mechanism by PVBV may also account for the increase in GAs in broom compared to PVBV-free, asymptomatic tissue. Possibly, eriophyid mite infestations of broom branches may lead to a decrease in GAs, as reported for *B. tabaci* and the tobacco aphid *Schlechtendalia chinensis* in Chinese Sumac (*Rhus chinensis*), and may add to the factors underlying GA dynamics in broom in relation to PVBV-free, asymptomatic, and adventitious shoots [119,120]. Cytokinin levels were the highest in PVBV-free, asymptomatic leaves, a response that may be correlated with seasonal effects, in that leaf growth and expansion are triggered by the monsoon season. The monsoon rains were particularly abundant during the summer of 2015, when these samples were collected, relative to other years. The relatively low levels of this hormone complex in PVBV-infected samples may be due to the virus, as documented for interactions between white clover mosaic (*Potexvirus*) and common bean plants [121]. Consistent with increased GA, the mean levels of cytokinin, an antagonist of GA, which is involved in cell division and expansion, were higher in leaves from broom compared to adventitious shoot samples [122].

Increased cytokinin production has been associated with colonization by other stylet-feeding insects such as *S. chinensis* in Chinese Sumac and *B. tabaci* in tobacco [119,120], which may explain the increased mean cytokinin levels in mite-infested broom leaves compared to broom-adventitious shoots where few mites were observed. According to heatmap and PCA biplots of primary metabolites, shikimic acid pathway metabolites were elevated in broom branch/leaves compared to asymptomatic adventitious shoots, even though they were collected from a virus-infected tree (Figure 8 and Figure S6A,B). This pathway produces auxin, which functions in cell elongation, as well as plant defense compounds containing aromatic amino acids, and phenolic compounds. Some plant viruses are known to regulate auxin/indole acetic acid (Aux/IAA) proteins, which are negative regulators of auxin, and negatively regulate auxin response factor (ARF) proteins, its positive regulators [123]. The enrichment of this biosynthesis pathway observed in broom leaves could be due to reduced auxin levels and/or to combat virus–mite dual infection in broom branches/leaves by the enhanced production of phenolic compounds.

In rose plants inoculated with an infectious clone of rose rosette virus, the virus presence alone was sufficient for disease development, in that mites were not intentionally introduced or known to be present on the inoculated plants [124]. Here, broom leaves showed increased chlorophyll metabolism and sugar alcohols (the latter, a product of the former), compared to virus-free and PVBV-infected asymptomatic adventitious shoots. In the PVBV–palo verde pathosystem, eriophyid mites appear to reside preferentially in broom-affected branches, where PVBV accumulation is highest among plant organs/tissues analyzed here, and either individually (one or the other) or synergistically trigger phytohormonal changes that lead to a localized increase in photosynthetic products (i.e., sugar alcohols). The extensive axillary bud growth associated with witches’ broom formation (e.g., hibiscus, *Paulownia*, Key lime, etc.) is common in plants infected by phytoplasmas [19,125,126]. In the absence of phytoplasma detected in BPV leaf tissue (authors), possibly elevated sugar levels in brooms associated with PVBV and/or mite presence may have simulated physiological changes potentially analogous to phytoplasma infection, e.g., by stimulating IAA, repressing auxin production, altering apical dominance, and ultimately enhancing axillary bud growth [127,128,129]. Potentially, interactions involving colonization by the suspect mite and/or PVBV infection alone or together also induce altered metabolic and hormonal changes in multiple plant organs/tissues. Additional studies are needed to better understand the biological basis of these putative, tritrophic interactions.

This is the first report of the full-length PVBV genome sequence and its characterization since the initial association of PVBV with witches’ broom disease of blue palo verde [9]. The small RNA analysis provides evidence for the activation of host defenses associated with PVBV-infected and apparently virus-free shoots, based on quantification and characterization of vsiRNAs. Although Koch’s postulates have not been demonstrated by grafting or mite-transmission, evidence of small RNAs of PVBV origin and elevated phytohormone levels in symptomatic/asymptomatic virus-infected trees compared to asymptomatic, virus-free trees (negative RT-PCR detection) provided robust evidence that PVBV is the causal agent of witches’ broom disease. Determining additional PVBV genome sequences for geographically representative isolates will facilitate the development of molecular assays with broad detection capabilities, expand the understanding of PVBV genome diversity, and lead to an improved understanding of the epidemiology to guide approaches for disease management.

## Figures and Tables

**Table 1:** Details of reference sequence mapping of total RNA-seq reads of the palo verde broom virus (PVBV) RNA 1–5 genome segments sequenced from different palo verde tree plant parts.

Isolate *	Total No. of Reads	No. of PVBV-Specific Reads (%)	No. of Reads Specific to Each RNA Segment (Percent Proportion of PVBV Specific Sequence Reads)				
			RNA1	RNA2	RNA3	RNA4	RNA5
**P2**	29,215,291	1431 (0.00%)	135 (9%)	146 (10%)	700 (49%)	355 (25%)	95 (7%)
**P3**	79,809,277	585,276 (0.73%)	88,963 (15%)	62,888 (11%)	259,148 (44%)	140,214 (24%)	34,063 (6%)
**P4**	50,523,789	1,286,718 (2.55%)	84,487 (7%)	56,810 (4%)	737,004 (57%)	321,399 (25%)	87,018 (7%)
**P5**	54,202,178	16,677 (0.03%)	1122 (7%)	1112 (7%)	9128 (55%)	4061 (24%)	1254 (4%)
**P8**	39,762,182	456,385 (1.15%)	69,973 (15%)	79,776 (17%)	205,677 (45%)	81,051 (18%)	19,908 (4%)
**P9**	59,815,606	464,972 (0.78%)	111,943 (24%)	77,841 (17%)	154,015 (33%)	70,033 (15%)	51,140 (11%)

* P2: leaves from asymptomatic trees; P3: leaves from symptomatic trees; P4: leaves from symptomatic trees; P5: adventitious shoots from symptomatic trees; P8: flowers from symptomatic trees; and P9: seeds from symptomatic trees.

**Table 2:** Percent pairwise nucleotide identity and amino acid similarity comparisons of the five RNA genome segments and predicted proteins encoded by the palo verde broom virus (PVBV) genome, with representative relatives in the genus *Emaravirus*.

Emaravirus *	Palo Verde Broom Virus Genome Segment and Coding Region **									
	RNA1	RdRp	RNA2	GP	RNA3	NP	RNA4	MP	RNA5	HP
**HPWMoV**	**62.5**	**50.8**	60.0	**40.6**	64.3	**35.2**	**63.7**	**51.7**	**64.5**	27.2
**CORSaV**	60.6	43.8	59.2	32.6	59.3	32.8	58.6	45.7	57.2	26.9
**TiRSaV**	62.3	43.5	61.0	32.7	**65.0**	32.8	63.4	50.5	65.3	24.4
**JYMaV**	59.2	44.0	58.1	35.3	61.5	33.6	58.0	45.5	62.0	19.5
**RLBV**	61.4	43.1	59.9	34.0	62.1	28.5	62.9	44.4	64.1	**29.3**
**AcCRaV**	60.7	33.8	58.7	28.4	60.1	22.9	61.0	24.7	62.8	15.2
**AcEV-2**	60.3	36.9	60.0	25.2	61.3	23.6	59.6	21.2	62.9	22.6
**AsMaV**	59.1	35.4	60.2	25.4	56.0	23.3	58.6	22.4	58.5	18.6
**BLMaV**	61.1	34.1	57.6	24.2	60.9	21.3	57.0	20.9	63.1	20.1
**ChMaV**	59.3	33.5	59.3	24.1	56.1	28.7	58.5	20.7	58.4	15.2
**CjaV-1**	61.2	31.6	59.1	24.7	61.5	24.9	58.5	28.8	61.6	20.9
**CjaV-2**	61.5	31.8	57.5	26.3	60.7	23.5	60.2	28.0	-	-
**EMARaV**	58.7	35.9	59.3	26.0	59.0	21.2	57.0	21.8	-	-
**FMV**	58.8	35.8	59.6	24.4	56.2	24.1	58.7	21.7	60.4	22.8
**KOPV**	60.1	33.8	**61.3**	21.4	61.9	24.4	59.0	26.2	63.1	19.5
**LiCRaV**	59.5	35.0	59.7	24.8	58.6	22.5	58.3	23.3	59.2	17.1
**MaMaV**	60.6	36.0	60.2	27.0	58.8	23.6	57.3	24.5	62.3	24.1
**PCLSaV**	60.3	32.7	59.1	22.8	59.0	24.4	58.8	24.4	57.4	15.5
**PerMV**	59.1	30.1	59.3	23.1	60.8	21.0	57.9	26.8	61.0	17.9
**PiVB**	60.4	35.7	58.9	25.1	59.0	24.3	56.8	22.6	60.1	27.8
**PPSMV-1**	60.7	36.3	59.4	25.2	54.4	26.0	58.4	20.8	62.4	24.9
**PPSMV-2**	59.0	35.8	60.1	24.5	56.1	21.8	57.5	22.5	63.0	27.6
**RRV**	61.0	35.8	59.6	24.6	63.8	23.3	60.7	23.0	62.6	22.8
**RYRSaV**	60.7	36.7	58.6	26.7	59.7	26.5	59.1	19.3	61.7	18.4
**VEV**	58.8	34.4	60.2	27.1	57.9	23.2	56.3	19.0	57.5	18.1

Legend: * HPWMoV: high plains wheat mosaic virus; CORSaV: common oak ringspot-associated virus; TiRSaV: ti ringspot-associated virus; JYMaV: jujube yellow mottle-associated virus; RLBV: raspberry leaf blotch virus; AcCRaV: actinidia chlorosis ringspot-associated virus; AcEV-2: actinidia virus 2; AsMaV: aspen mosaic-associated virus; BLMaV: blackberry leaf mottle-associated virus; ChMaV: chrysanthemum mosaic-associated virus; CjaV-1: camellia japonica associated virus 1; CjaV-2: camellia japonica associated virus 2; EMARaV: European mountain ash ringspot-associated virus; FMV: fig mosaic virus; KŌPV: Karaka Ōkahu purepure virus; LiCRaV: lilac chlorotic ringspot-associated virus; MaMaV: maple mottle-associated virus; PCLSaV: pear chlorotic leaf spot-associated virus; PerMV: perilla mosaic virus; PiVB: Pistacia emaravirus; PPSMV-1: pigeonpea sterility mosaic virus 1; PPSMV-2: pigeonpea sterility mosaic virus 2; RRV: rose rosette virus; RYRaV: redbud yellow ringspot-associated virus; VEV: vitis emaravirus. ** RNA segment with predicted coding region function; RdRp: RNA-dependent RNA polymerase; GP: glycoprotein; NP: nucleocapsid protein; MP: movement protein; HP: hypothetical protein.

**Table 3:** Number of reads and percentage proportion of small interfering RNA sequence reads mapped to the genome segments of palo verde broom virus (PVBV) associated with each plant part and/or organ. **Legend:** * P2: leaves from asymptomatic trees, P3: leaves from symptomatic trees, P4: leaves from symptomatic trees, P5: adventitious shoots from symptomatic trees, P8: flowers produced by symptomatic trees, and P9: seeds produced by symptomatic trees. * cpm: counts per million.

Isolate *	Total No. Reads	No. PVBV-Specific Reads (cpm) *	No. of Reads Specific to Each PVBV RNA Genome Segment in Counts Per Million				
			RNA1	RNA2	RNA3	RNA4	RNA5
**P2**	29,624,358	382.00 (0.00%)	24.98 (6%)	25.49 (7%)	268.26 (70%)	17.27 (5%)	46.01 (12.0%)
**P3**	18,673,896	197,065.36 (1.05%)	12,163.66 (6%)	9185.44 (5%)	99,072.58 (50%)	66,541.34 (34%)	10,102.34 (5%)
**P4**	16,049,539	216,875.39 (1.35%)	13,375.59 (6%)	10,324.16 (5%)	114,104.52 (53%)	57,637.17 (26%)	21,433.95 (10%)
**P5**	11,889,372	70,122.80 (0.59%)	3349.97 (5%)	4373.23 (6%)	32,991.90 (47%)	25,880.42 (37%)	3527.27 (5%)
**P8**	9,194,688	110,337.29 (1.20%)	7457.89 (7%)	20,365.67 (18%)	51,950.76 (47%)	26,989.93 (25%)	3573.04 (3%)
**P9**	13,380,230	77,302.41 (0.58%)	3166.46 (4%)	8220.34 (11%)	41,403.25 (53%)	17,826.82 (23%)	6685.54 (9%)

## Data Availability

The nucleotide sequences reported in this study have been deposited in the NCBI GenBank database at https://www.ncbi.nlm.nih.gov/.

## Supplementary Materials

The following supporting information can be downloaded at: https://www.mdpi.com/article/10.3390/v17081122/s1, **Figure S1**: Prediction of transmembrane helices in the glycoprotein of PBV (A) and prediction of signal peptides in the glycoprotein (B); **Figure S2**: Amino acid sequence alignment and prediction of protein secondary structure for the P4 protein of palo verde broom virus (PVBV) and P4 proteins of closely related emaravirus orthologs. The alignment shows the underlined core domain similar to the 30 K superfamily of viral movement proteins. The first line in each block shows the conservation indices for positions with a conservation index above 5. Representative sequences indicated in magenta letters are colored according to predicted secondary structures (red: h; alpha-helix, blue: e; beta-strand). The sequences indicated in black letters directly under a representative sequence are in the same pre-aligned group. Consensus predicted secondary structure symbols: alpha-helix: h; beta-strand: e. Consensus amino acid symbols—conserved amino acids are in bold and uppercase letters; aliphatic (I, V, L): l; aromatic (Y, H, W, F): @; hydrophobic (W, F, Y, M, L, I, V, A, C, T, H): h; alcohol (S, T): o; polar residues (D, E, H, K, N, Q, R, S, T): p; tiny (A, G, C, S): t; small (A, G, C, S, V, N, D, T, P): s; bulky residues (E, F, I, K, L, M, Q, R, W, Y): b; positively charged (K, R, H): +; negatively charged (D, E): -; and charged (D, E, K, R, H): c. The last two lines show consensus amino acid sequences (Consensus_aa) and consensus predicted secondary structures (Consensus_ss). The analysis was carried out using PROMALS3D [69]. Palo verde broom virus (PVBV), high plains wheat mosaic virus (HPWMoV), common oat ringspot-associated virus (CORSaV), ti ringspot-associated virus (TiRSaV), raspberry leaf blotch virus (RLBV), and jujube yellow mottle-associated virus (JYMaV); **Figure S3**: Sequence length distribution profiles of small RNA sequences from palo verde samples (A) P2: asymptomatic trees; (B) P3: leaves from symptomatic trees, (C) P4: leaves from symptomatic trees, (D) P5: leaves from adventitious shoots of symptomatic trees, (E) P8: flowers from symptomatic tree, and (F) P9: seeds from symptomatic trees; **Figure S4**: Strand bias plot of small RNA profiles of palo verde samples (A) P2: asymptomatic trees, (B) P3: leaves from symptomatic trees, (C) P4: leaves from symptomatic trees, (D) P5: leaves from adventitious shoots of symptomatic trees, (E) P8: flowers from symptomatic tree, and (F) P9: seeds from symptomatic trees; **Figure S5**: The 20–24 nt siRNA target sites and abundance (counts per million) in the PVBV genomic segments. P3 and P4: leaves from symptomatic trees (A through E), P5: leaves from adventitious shoots of symptomatic trees (F through J), P8: flowers from symptomatic tree (K through O), and P9: seeds from symptomatic trees (P through T); **Figure S6**: PCA biplot of primary metabolites (A); PCA biplot of primary metabolites re-labeled according to functional categories (B); **Table S1**: Virus-specific primers used for RT-PCR detection of palo verde broom virus (PVBV) RNA segments in PVBD-symptomatic samples; **Table S2**: Confirmation of palo verde broom virus (PVBV) infection in symptomatic blue palo verde trees.
