# Supplementary material for: First Complete Genome Sequence of Palo Verde Broom Emaravirus, Virus-Derived siRNA Signatures, and Phytohormone-Metabolite Profiling of Witches’ Broom-Affected Palo Verde Trees
Source: Viruses. 2025 Aug 15;17(8):1122. doi: 10.3390/v17081122 (PMC12390736; doi:10.3390/v17081122)
Supplement: Supplementary file 1 [file viruses-17-01122-s001.zip › viruses-3751541-supplementary.pdf]

Supplementary Table S1. Virus-specific primers used for RT-PCR detection of Palo verde broom virus (PVBV) RNA segments in PVBD-symptomatic samples.

Genome segment	Primer	Sequence	Tm (°C)[1]	Amplicon length (bp)[1]
**RNA1**	PVRNA1_1892F	5′ GGTTGTTATTGAATATCTACCATACTC 3′	55	1127
	PVRNA1_3018R	5′ TATCAACACTAATAATAGGTAATGAGC 3′		
**RNA2**	PVRNA2_455F	5′ TCTGCTTCCGTGTTGTATTGA 3′	55	639
	PVRNA2_1093R	5′ AAGTGTCTATGGTGTTGTTGATG 3′		
**RNA3**	PVRNA3_433F	5′ ACTCTCCTGTCTTCTCTTCATCA 3′	56	679
	PVRNA3_1111R	5′ GTCCGTAGCATTGACTGTGA 3′		
**RNA4**	PVRNA4_339F	5′ GCCTCATCCTCTTTCCTTTCA 3′	56	754
	PVRNA4_1092R	5′ ACAAGCCACATACTGACAAATGC 3′		
**RNA5**	PVRNA5_440F	5′ TAGGTTCACCAGCTCAAGGC 3′	55	597
	PVRNA5_1015R	5′ GCATCATCAGCACAAGRACCAC 3′		

[1] Tm: annealing temperature; bp: base pair.

Supplementary Table S2. Confirmation of palo verde broom virus (PVBV) infection in symptomatic blue palo verde trees.

Isolate[1]	PVBV genome segment	Primers used for PCR	Amplicon size (bp)	GenBank accession no.
**OM2**	RNA 1	PVRNA1_1892F & PVRNA1_3018R	1127	OM273626
	RNA 2	PVRNA2_455F & PVRNA2_1093R	639	OM273627
	RNA 3	PVRNA3_433F & PVRNA3_1111R	679	OM273628
	RNA 4	PVRNA4_339F & PVRNA4_1092R	754	OM273629
	RNA 5	PVRNA5_440F & PVRNA5_1015R	597	OM273630
**SSE**	RNA 1	PVRNA1_1892F & PVRNA1_3018R	1127	OM273631
	RNA 2	PVRNA2_455F & PVRNA2_1093R	639	OM273632
	RNA 3	PVRNA3_433F & PVRNA3_1111R	679	OM273633
	RNA 4	PVRNA4_339F & PVRNA4_1092R	754	OM273634
	RNA 5	PVRNA5_440F & PVRNA5_1015R	597	OM273635
**BPV18-GH**	RNA 1	PVRNA1_1892F & PVRNA1_3018R	1127	OM273636
	RNA 2	PVRNA2_455F & PVRNA2_1093R	639	OM273637
	RNA 3	PVRNA3_433F & PVRNA3_1111R	679	OM273638
	RNA 4	PVRNA4_339F & PVRNA4_1092R	754	OM273639
	RNA 5	PVRNA5_440F & PVRNA5_1015R	597	OM273640

[1] Isolate source: OM2: Old Main 2, SSE: Social Science East, BPV18-GH: Sixth street garage greenhouse.

(A)

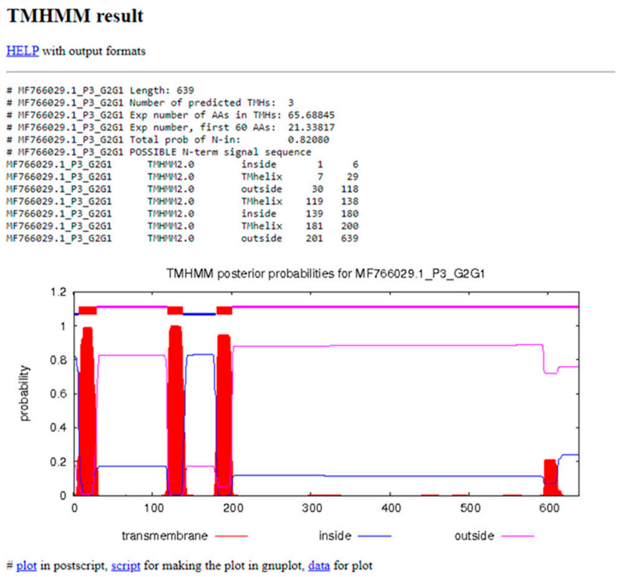

(B)

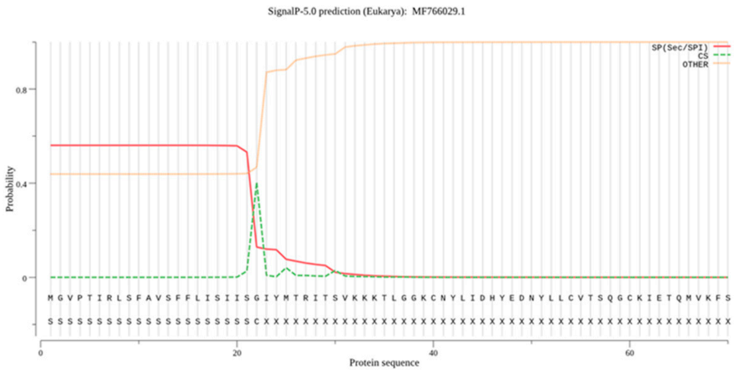

Supplementary Figure S1. Prediction of transmembrane helices in the glycoprotein of PBV (A.), and prediction of signal peptides in the glycoprotein (B.).

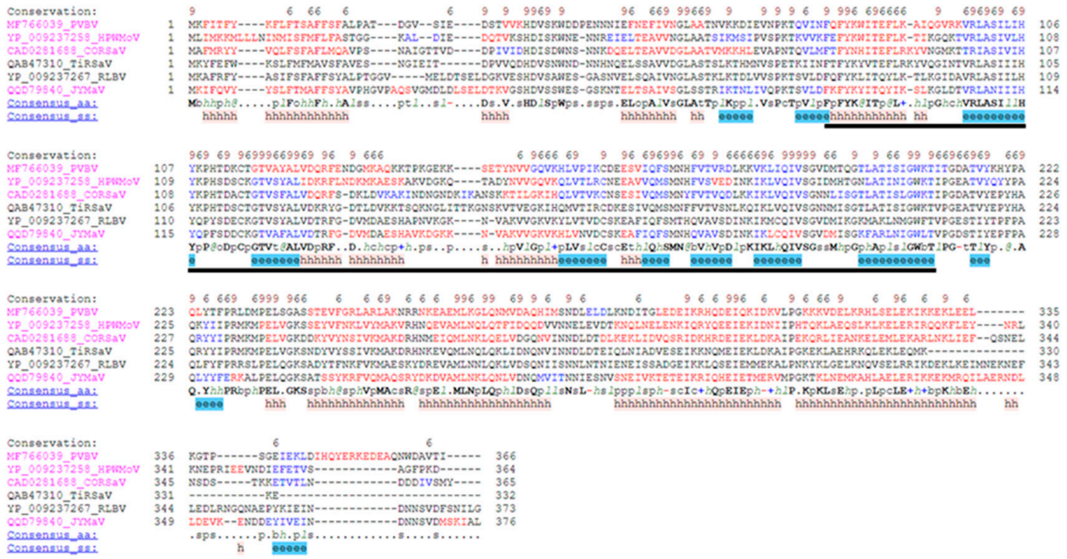

Supplementary Figure S2. Amino acid sequence alignment and prediction of protein secondary structure for the P4 protein of palo verde broom virus (PVBV) and P4 proteins of closely related emaravirus orthologs. The alignment shows the underlined core domain similar to the 30K superfamily of viral movement proteins. The first line in each block shows the conservation indices for positions with a conservation index above 5. Representative sequences indicated in magenta letters are colored according to predicted secondary structures (red: h; alpha-helix, blue: e; beta-strand). The sequences indicated in black letters directly under a representative sequence are in the same pre-aligned group. Consensus predicted secondary structure symbols: alpha-helix: h; beta-strand: e. Consensus amino acid symbols are: conserved amino acids are in bold and uppercase letters; aliphatic (I, V, L): l; aromatic (Y, H, W, F): @; hydrophobic (W, F, Y, M, L, I, V, A, C, T, H): h; alcohol (S, T): o; polar residues (D, E, H, K, N, Q, R, S, T): p; tiny (A, G, C, S): t; small (A, G, C, S, V, N, D, T, P): s; bulky residues (E, F, I, K, L, M, Q, R, W, Y): b; positively charged (K, R, H): +; negatively charged (D, E): -; charged (D, E, K, R, H): c. The last two lines show consensus amino acid sequence (Consensus_aa) and consensus predicted secondary structures (Consensus_ss). The analysis was carried out using PROMALS3D (Pei and Grishin., 2007). Palo verde broom virus (PVBV), high plains wheat mosaic virus (HPWMoV), common oat ringspot-associated virus (CORSaV), ti ringspot-associated virus (TiRSaV), raspberry leaf blotch virus (RLBV), and jujube yellow mottle-associated virus (JYMaV).

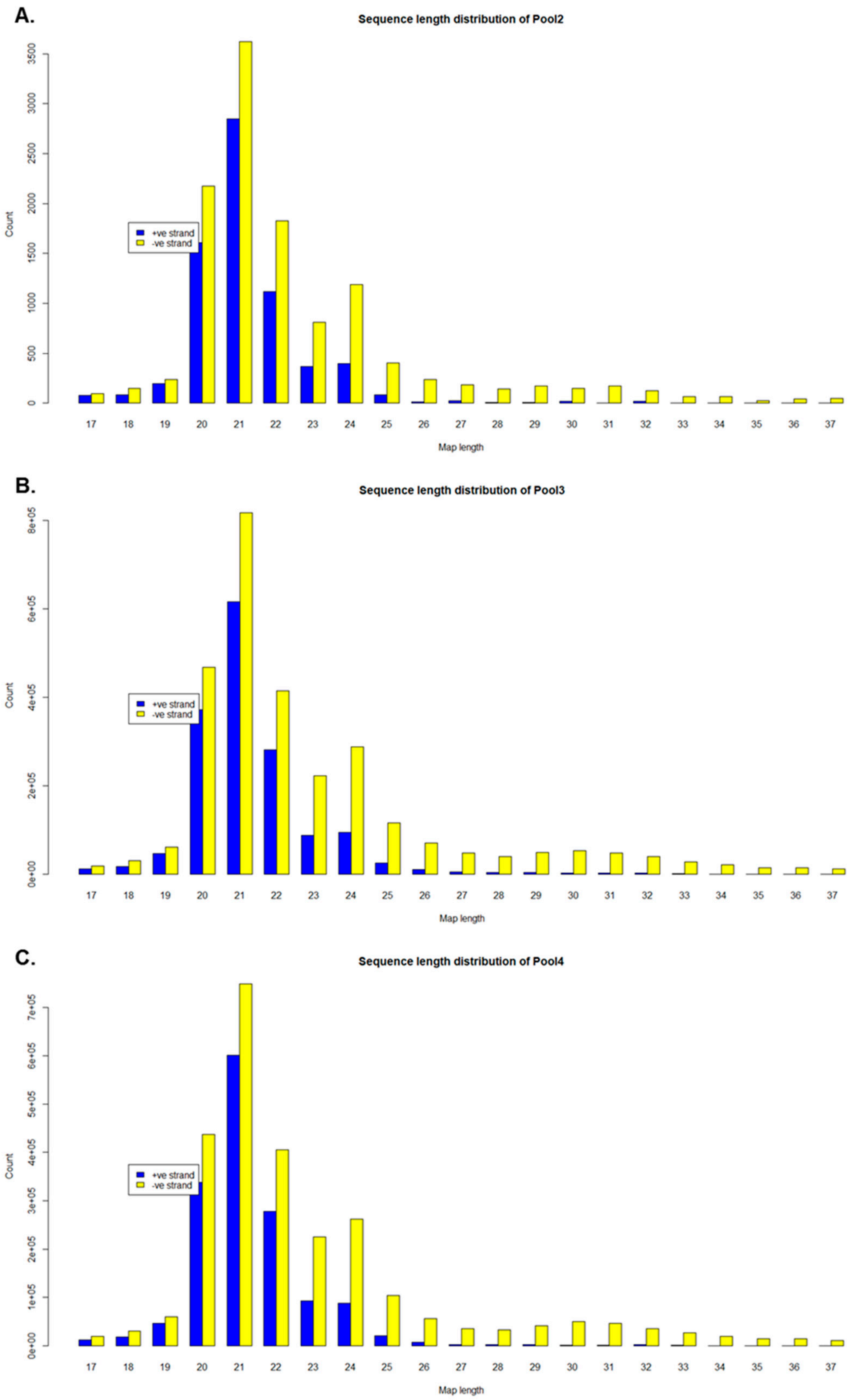
A.
Sequence length distribution of Pool2
Count
+ve strand
-ve strand
Map length
B.
Sequence length distribution of Pool3
Count
+ve strand
-ve strand
Map length
C.
Sequence length distribution of Pool4
Count
+ve strand
-ve strand
Map length

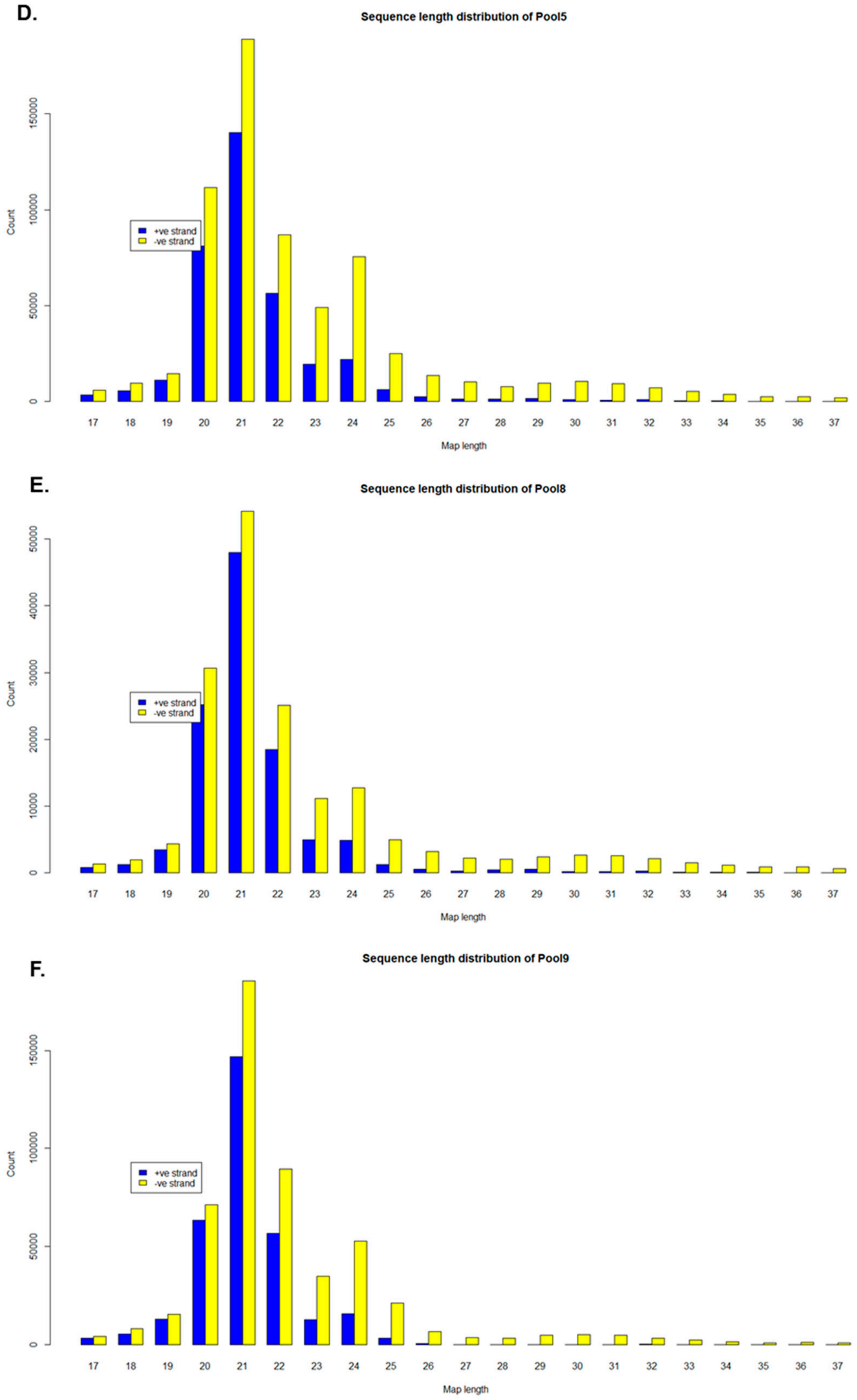

Supplementary Figure S3. Sequence length distribution profiles of small RNA sequences from palo verde samples (A.) P2: asymptomatic trees, (B.) P3: leaves from symptomatic trees, (C.) P4: leaves from symptomatic trees, (D.) P5: leaves from adventitious shoots of symptomatic trees, (E.) P8: flowers from symptomatic tree, and (F.) P9: seeds from symptomatic trees.

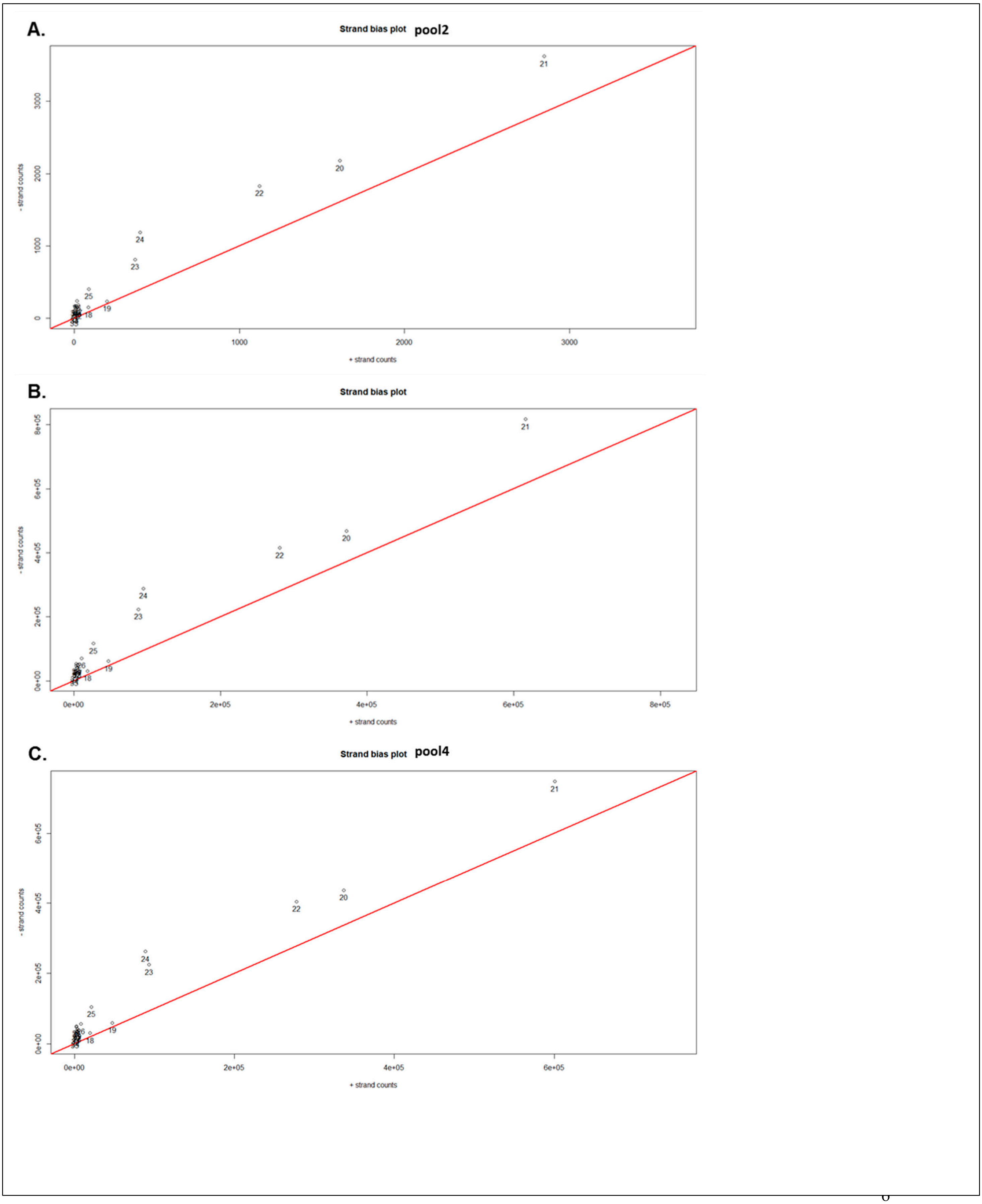

A.
Strand bias plot pool2
- strand counts
+ strand counts
B.
Strand bias plot
- strand counts
+ strand counts
C.
Strand bias plot pool4
- strand counts
+ strand counts

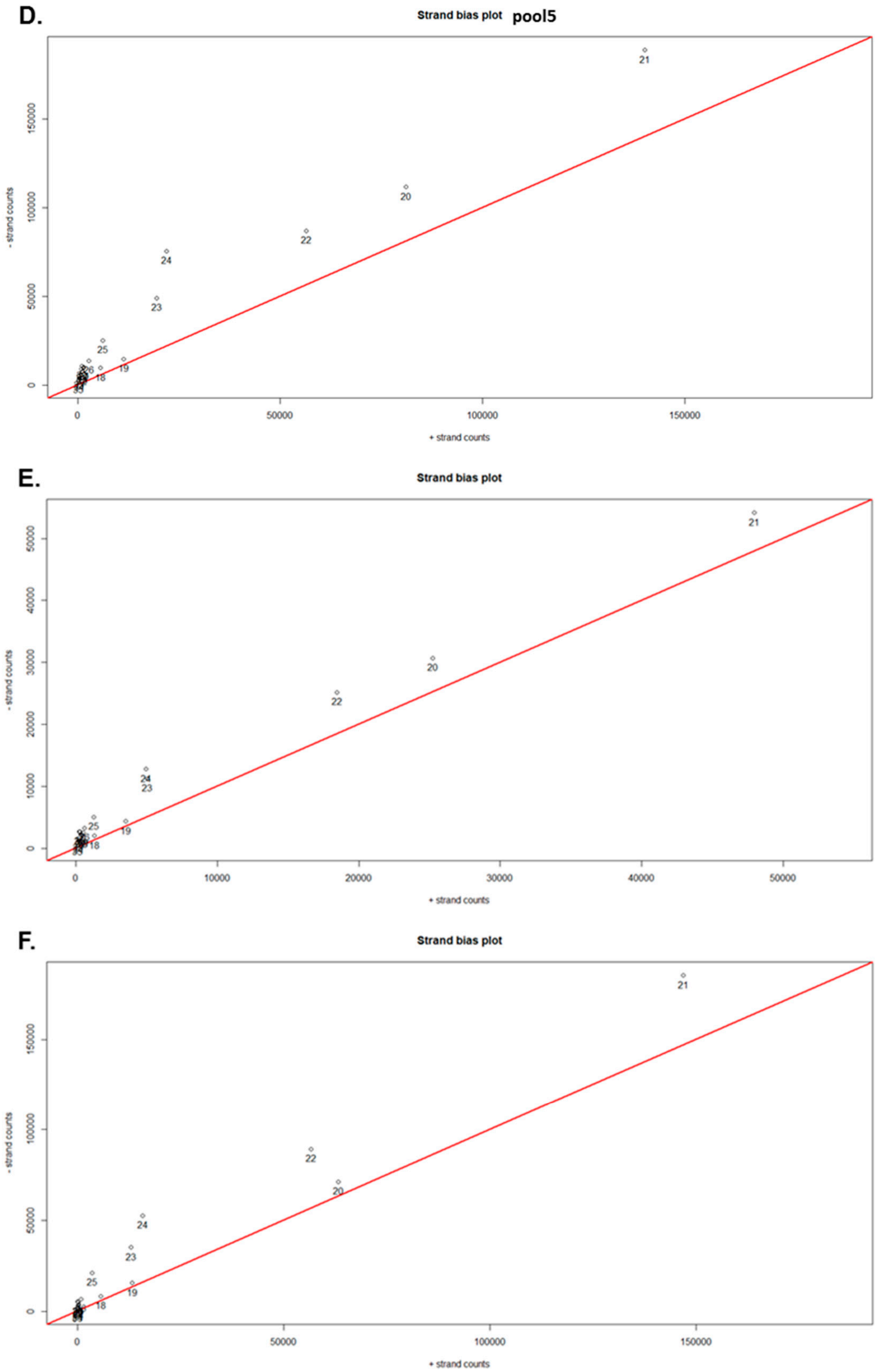

Supplementary Figure S4. Strand bias plot of small RNA Profiles of palo verde samples (A.) P2: asymptomatic trees, (B.) P3: leaves from symptomatic trees, (C.) P4: leaves from symptomatic trees, (D.) P5: leaves from adventitious shoots of symptomatic trees, (E.) P8: flowers from symptomatic tree, and (F.) P9: seeds from symptomatic trees.

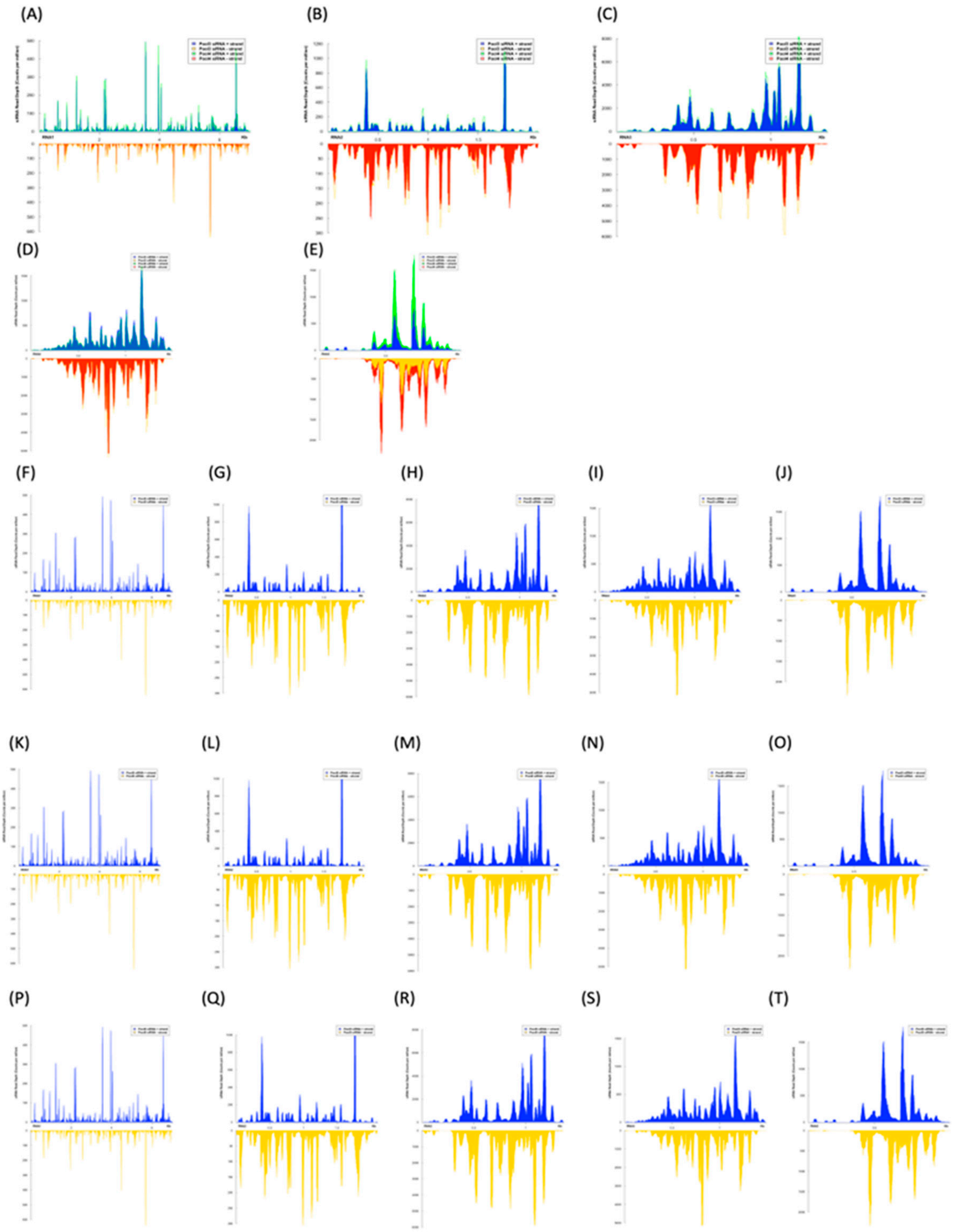

Supplementary Figure S5. The 20-24nt siRNA target sites and abundance (counts-per-million) in the PVBV genomic segments. P3 and P4: leaves from symptomatic trees (A through E), P5: leaves from adventitious shoots of symptomatic trees (F through J), P8: flowers from symptomatic tree (K through O), and P9: seeds from symptomatic trees (P through T).

(A)

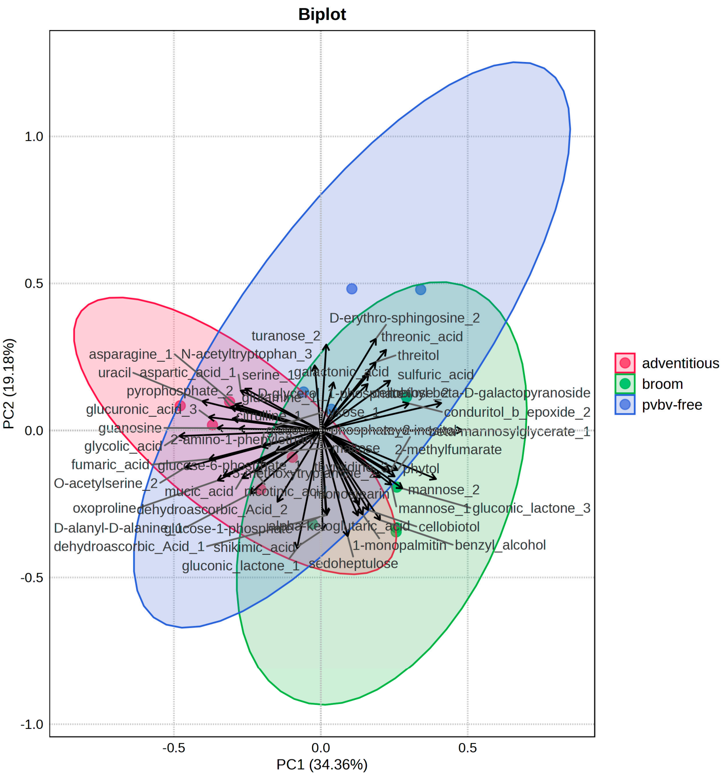

(B)

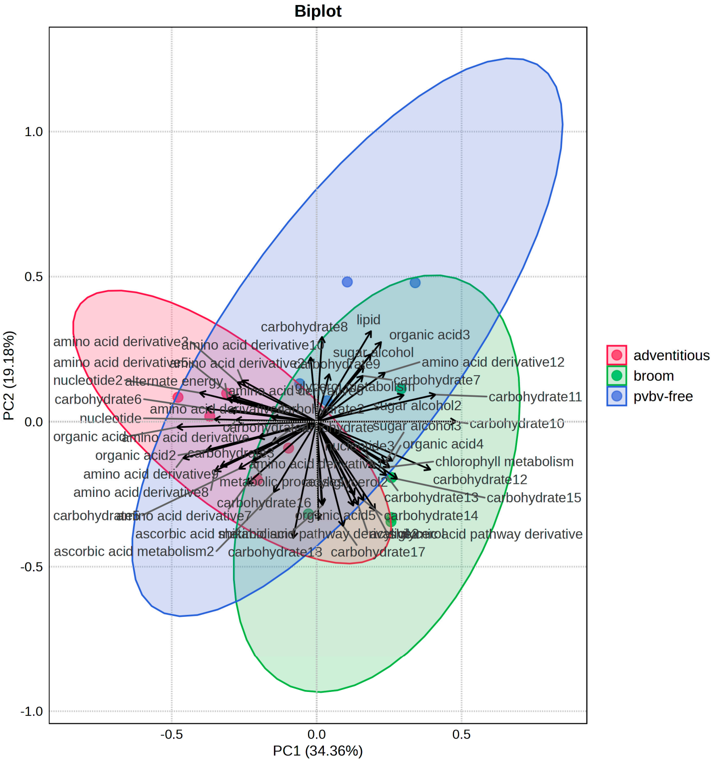

Supplementary Figure S6. PCA biplot of primary metabolites (A), and corresponding functional categories (B) in leaves from PVBV-free, asymptomatic trees, PVBV-infected, symptomatic (broom), and PVBV-infected, asymptomatic (adventitious) shoots.
